# Impact of Exercise Training in Patients with Diabetic Peripheral Neuropathy: An Umbrella Review

**DOI:** 10.1186/s40798-025-00863-4

**Published:** 2025-06-15

**Authors:** Alba Gracia-Sánchez, Adriana López-Pineda, Rauf Nouni-García, Sara Zúnica-García, Esther Chicharro-Luna, Vicente F. Gil-Guillén

**Affiliations:** 1 Behavioral Sciences and Health Department, Nursing Area, Faculty of Medicine, Miguel Hernandez University, San Juan de Alicante, Spain; 2 Clinical Medicine Department, Miguel Hernandez University, Ctra. Nnal. 332 S/N 03550, San Juan de Alicante, Spain; 3 Network for Research on Chronicity, Primary Care, and Health Promotion (RICAPPS), San Juan de Alicante, Alicante, Spain; 4 Pathology and Surgery Department, Miguel Hernandez University, San Juan de Alicante, Spain; 5 Institute of Health and Biomedical Research of Alicante (ISABIAL), Alicante, Spain; 6 Research Unit, University General Hospital of Elda, Elda, Spain

**Keywords:** Diabetic peripheral neuropathy, Exercise, Balance, Falls, Systematic review, Diabetes

## Abstract

**Background:**

Diabetic peripheral neuropathy (DPN) is a common and serious complication of diabetes mellitus, affecting sensory, motor, and autonomic nerves. It increases the risk of foot ulceration and falls. Management typically involves preventive strategies like patient education, risk stratification, and regular foot screenings. Exercise plays a key role in enhancing glycemic control and nerve function, reducing the risk of DPN and related complications. This umbrella review aimed to evaluate the impact of different exercise interventions on patients with DPN.

**Methods:**

The search was conducted in the following databases: Pubmed, Scopus, Cochrane Library, Embase, and SPORTDiscus, from the establishment of the database up to the search date (September 11, 2023). We included systematic reviews and meta-analyses assessing exercise interventions in adults with type 1 or type 2 diabetes and DPN. Studies were selected based on predefined PICO criteria. The methodological quality of included reviews was assessed using the AMSTAR-2 tool. Results were synthesized narratively and categorized by exercise type and health outcome.

**Results:**

Fourteen reviews were included, examining the effects of various exercise interventions. Duration ranged from one week to 12 months, and studies were conducted in multiple countries. Additionally, we extracted and reanalyzed individual results from 70 primary studies included within the reviews. Some meta-analyses reported significant improvements in fasting glucose and HbA1c (n = 1), neuropathic symptoms (n = 3), physical function (n = 1), static and dynamic balance (n = 2), range of motion (n = 1), and fear of falling (n = 1). No significant effects were found for BMI, ulcer incidence, adverse events, weight-bearing activity, quality of life, or forefoot plantar pressure (n = 1). Outcome assessment tools included the Biodex system (n = 9), single-leg stance (n = 8), Berg Balance Scale (n = 11), and Timed Up and Go (n = 13) for balance; nerve conduction velocity (n = 8), MNSI (n = 6), and Total Symptom Score (n = 3) for nerve function; fasting glucose (n = 3) and HbA1c (n = 5) for glycemic control. Other outcomes included muscle strength (n = 6), functional capacity (n = 6), pain (n = 6), and quality of life (n = 6).

**Conclusions:**

Exercise training appears to have potential benefits for certain aspects of DPN, neuropathic symptoms, and functional capacity. However, the effects on glycemic control, fall risk reduction, and ulcer prevention remain inconclusive, with significant variability in study outcomes.

**Supplementary Information:**

The online version contains supplementary material available at 10.1186/s40798-025-00863-4.

## Key points

- Exercise interventions, including aerobic, resistance, and balance training, show beneficial effects on neuropathic symptoms, nerve conduction, glycemic control, and postural stability in patients with diabetic peripheral neuropathy.
- Combined aerobic and resistance training appears more effective than single-mode interventions in reducing HbA1c and improving metabolic outcomes
- The substantial heterogeneity in study designs and exercise protocols underscores the need for standardized, evidence-based exercise guidelines tailored to this population

**Supplementary Information:**

The online version contains supplementary material available at 10.1186/s40798-025-00863-4.

## Background

Diabetes mellitus is a group of metabolic disorders characterized by hyperglycemia due to defects in insulin secretion and/or action. According to the American Diabetes Association (ADA), it is classified into type 1 (T1DM) and type 2 diabetes (T2DM) [1]. T1DM, typically diagnosed in children and young adults, results from autoimmune destruction of pancreatic β-cells and leads to absolute insulin deficiency [1]. In contrast, T2DM is the most common form and is marked by insulin resistance and progressive β-cell dysfunction, mainly affecting adults, although its incidence is rising among younger populations in Western countries due to obesity and sedentary lifestyles [2]. In some populations, such as Asian populations, T2DM can develop in individuals with a lower body mass index (BMI) compared to Western populations [3]. Globally, T2DM represents a growing public health concern [4, 5], with the number of adults with diabetes expected to rise from 536.6 million in 2021 to 783.2 million by 2045 [3]. Additionally, the clinical presentation of T2DM varies by population, with significant differences in obesity prevalence and metabolic risk factors among different geographic regions [3]. It has also been reported that adults with diabetes are 1.55 times more likely to develop sarcopenia than those without diabetes [6].

One of the most common complications of diabetes is diabetic peripheral neuropathy (DPN), which affects both T1DM and T2DM patients. Epidemiological studies have reported that DPN prevalence ranges from 6 to 51%, depending on the population studied [7–10]. DPN is a key factor in the development of diabetic foot ulcers, making it the leading cause of non-traumatic lower limb amputations in high-income countries [11, 12]. More than 50% of DPN patients experience intense neuropathic pain, interfering with daily activities and significantly reducing their quality of life [13]. Additionally, DPN is associated with neuromuscular impairments and sensorimotor deficits, increasing the risk of falls and functional disabilities [14]. A meta-analysis reported that patients with diabetes are 1.55 times more likely to develop sarcopenia than non-diabetic individuals, further exacerbating functional decline [6].

Physical exercise is a fundamental component of diabetes management, with well-established benefits for glycemic control, cardiovascular health, body composition, muscle strength, and overall functional capacity [15, 23, 24]. However, in patients with T1DM, its application requires careful management to prevent hypoglycemia, as tight glucose regulation is essential during and after exercise [16, 17]. Despite these challenges, exercise contributes to improvements in metabolism, muscle mass, and cardiorespiratory fitness in this population [19].

The ADA recommends at least 150 min of moderate-to-vigorous aerobic exercise per week, spread over at least three days with no more than two consecutive rest days, along with 2–3 sessions of resistance training per week [15]. A recent meta-analysis highlighted that long-term exercise programs, especially in children and adolescents with T1DM, yield significant improvements in BMI, glycemic markers, and physical fitness [19].

Beyond physiological outcomes, T1DM patients often report lower quality of life compared to those with T2DM, due to a greater burden of comorbidities, accelerated functional decline with aging, and reduced life satisfaction [18]. This underscores the importance of comprehensive approaches to diabetes care, including both exercise and patient education. In fact, diabetes self-management education has proven to be a key strategy for improving disease control [4, 20, 21].

The International Diabetes Federation (IDF) recommends glycemic targets of fasting plasma glucose < 7.0 mmol/L and HbA1c < 7% [22], but poor glycemic control remains common and contributes to the development of complications such as DPN. In this context, therapeutic exercise, defined as structured and individualized physical activity designed to achieve specific health goals, can be particularly beneficial. It includes aerobic, resistance, sensorimotor, and mobility training, and has shown promise in improving multiple outcomes in patients with neuropathic complications [25].

Previous systematic reviews by the International Working Group on the Diabetic Foot [15, 16] have reported weak but favorable results on the effects of foot-related and mobility exercises in increasing the range of motion of the foot and ankle, but less so in reducing plantar pressure points (PPPs). Various research studies have explored the impact of exercise training on DPN, examining different types of exercises, from aerobic and strength training to balance and flexibility exercises [17, 18]. These studies have encompassed diverse populations, including patients of different ages and levels of DPN severity, using various impact measures such as balance, quality of life, and pain reduction [19, 20]. However, there are controversies in the literature; for example, while some studies indicate significant improvements in nerve function and pain [17, 21], others show no relevant changes [22].

Although numerous systematic reviews have addressed the effects of exercise in patients with DPN, they vary considerably in terms of inclusion criteria, methodological quality, and the types of interventions and outcomes assessed. This heterogeneity makes it difficult to draw consistent conclusions and limits their applicability in clinical practice. Therefore, a comprehensive umbrella review is needed to synthesize and organize the available evidence, clarify which types of exercise are most effective for specific outcomes, and identify gaps that should guide future research and clinical recommendations. The primary aim of this systematic review was to assess the impact of exercise training interventions on any objectively measured health-related outcome in patients with DPN through an umbrella review. The secondary aim was to identify the outcome measures used to evaluate the benefits of exercise training and the tools employed for these assessments.

## Methods

This umbrella review was conducted following the methodological guidelines for umbrella reviews from the Joanna Briggs Institute [23]. The review protocol was previously registered in PROSPERO (CRD42022376467) [24], and the results have been reported in accordance with the PRISMA (Preferred Reporting Items for Systematic Reviews and Meta-Analyses) guidelines [25]. The following modifications were made from the initially registered protocol: information was extracted from each individual study, excluding those that did not meet the eligibility criteria, and included in the results synthesis in a tabulated format.

### Eligibility Criteria

The eligibility criteria were based on the PICOS structure of the following research question: What impact do different exercise training interventions on any health outcome have on patients with DPN?

- Population: Reviews that included studies with adult individuals (≥ 18 years) with type 1 or T2DM and DPN, regardless of sex, setting, and health status.
- Intervention/Exposure: Reviews that included studies analyzing aerobic or anaerobic exercise (defined as any protocolized intervention involving planned, structured, and repetitive voluntary bodily movements) as the intervention or exposure.
- Comparison: Reviews that included studies without a comparison group, as well as studies comparing with another intervention, usual clinical practice, or a non-exposed control group.
- Outcomes: Health changes that exercise training interventions provides to patients with DPN (e.g., improvement in nerve conduction, balance enhancement, pain reduction, improved mobility, or others), measured through various scales or questionnaires that allow quantification of the changes that may occur at the end of the intervention.
- Study Design: Systematic reviews with or without meta-analyses were considered. The studies included in the selected systematic reviews had to consider retrospective and prospective cohort studies, quasi-experimental studies, or experimental studies.

Exclusions were applied to reviews including articles that were not conducted on humans or populations with prior muscle atrophy due to neurological disorders or other causes, such as bedridden patients, or those with muscle atrophy due to an underlying pathology (e.g., cancer, HIV/AIDS, morbid obesity, malnutrition). Reviews with studies that did not have clear outcome measures, those without available full information or text, studies presented at conferences without full articles available, and any non-systematic reviews were also excluded. Additionally, only articles published in peer-reviewed journals were included; thus, reviews for theses, dissertations, conference proceedings, or trial registries were not considered.

### Information Sources

The search was conducted in the following databases: Pubmed, Scopus, Cochrane Library, Embase, and SPORTDiscus, from the establishment of the database up to the search date (September 11, 2023).

### Search Strategy

The search strategy for each database was developed using keywords that defined the population and intervention, combining controlled vocabulary with free-text terms using Boolean operators (Supplementary Material 1). There were no restrictions on language or publication dates, and only publication type was filtered.

### Selection Process

Once the search was completed in all databases, all identified records were incorporated into the COVIDENCE® software. After automatically removing duplicates, two reviewers independently screened the titles and abstracts to select potentially eligible studies for inclusion, and a third reviewer resolved discrepancies. The studies selected in this initial screening were then retrieved in full text and independently reviewed by the same two reviewers to select those that met the eligibility criteria. Discrepancies were resolved by a third reviewer. Articles that were not freely accessible were requested from the institutional library or the authors.

### Data extraction Process and Data Items

Four investigators extracted data from the finally included reviews using a predefined data extraction form in Microsoft Excel. Any disagreements were resolved through discussion. The extracted information of interest from each review included descriptive characteristics and the conclusion of the review:

1. First author and year of publication of the systematic review.
2. Study population included in the reviewed studies.
3. Type of exercise training considered eligible for inclusion in the studies by the review.
4. Comparison considered eligible for inclusion in the studies by the review.
5. Outcome variables considered eligible for inclusion in the studies by the review.
6. Databases used for the search.
7. Search period.
8. Number of studies included in the review and cumulative sample size.
9. Types of studies included in the review.
10. Country of origin of the studies included in the review.
11. Publication period of the studies included in the review.
12. Instruments used to assess the risk of bias in the studies.
13. Age of the population and sex distribution: average age or age range of participants and percentage of women in the studies included in the review.
14. Indication of whether a meta-analysis was performed.
15. Conclusion of the review authors.

Additionally, details of the population, intervention/exposure, and results of the individual studies included in the systematic reviews, which met the eligibility criteria of this umbrella review, were extracted:

16. Study population.
17. Intervention/exposure studied.
18. Comparator group(s).
19. Duration of the intervention/exposure.
20. Frequency of the intervention/exposure.
21. Outcome variables measured in the included studies and measurement methods.
22. Changes observed in the primary outcome variables at the end of the study.
23. Outcome variables for which a meta-analysis was performed and the number of studies included in the meta-analysis, if conducted.
24. Reported overall effect measures and heterogeneity measures, if a meta-analysis was conducted.

### Assessment of Risk of Bias in Included Studies

The assessment of the risk of bias in the included reviews was conducted using the AMSTAR-2 tool [26] which is designed for the critical appraisal of systematic reviews and meta-analyses and contains 16 items, seven of which are critical domains (items 2, 4, 7, 9, 11, 13, and 15). We rated the methodological quality of the reviews according to the following criteria: ‘high’ when there was one or no non-critical weakness; ‘moderate’ if there were more than one non-critical weakness; ‘low’ if there was one critical flaw with or without non-critical weaknesses; and ‘very low’ if there were more than one critical flaw with or without non-critical weaknesses. Two reviewers independently assessed the risk of bias of the reviews, and disagreements were resolved through discussion between them.

### Synthesis Method

The characteristics of each review were presented in tables. The conclusions of each included review were examined to ensure that the information provided in the review was accurately represented, and when necessary, additional information was added by the authors of this review to provide relevant data that would help answer the research question posed in this umbrella review. However, given the generally low methodological quality of the reviews (as assessed using AMSTAR-2), and the heterogeneity in the types of interventions and outcomes reported, we opted to extract and synthesize data of the interventions and results directly from the individual primary studies that met our eligibility criteria. This allowed us to provide a more consistent and transparent qualitative synthesis, minimizing the influence of reporting flaws in the reviews themselves. Interventions were grouped into categories based on conditioning capacities.

Additionally, a table was included that listed the evaluated outcomes and the tools used, along with the frequency of use, i.e., the number of studies in which those tools were employed. A table was created to categorize the studies according to the type of exercise and indicate whether they had a positive effect on the most studied outcomes. Finally, a table with the results of the meta-analyses conducted in the reviews was presented. Meta-analysis was not considered due to the great variability of the interventions.

## Results

**Fig. 1:**
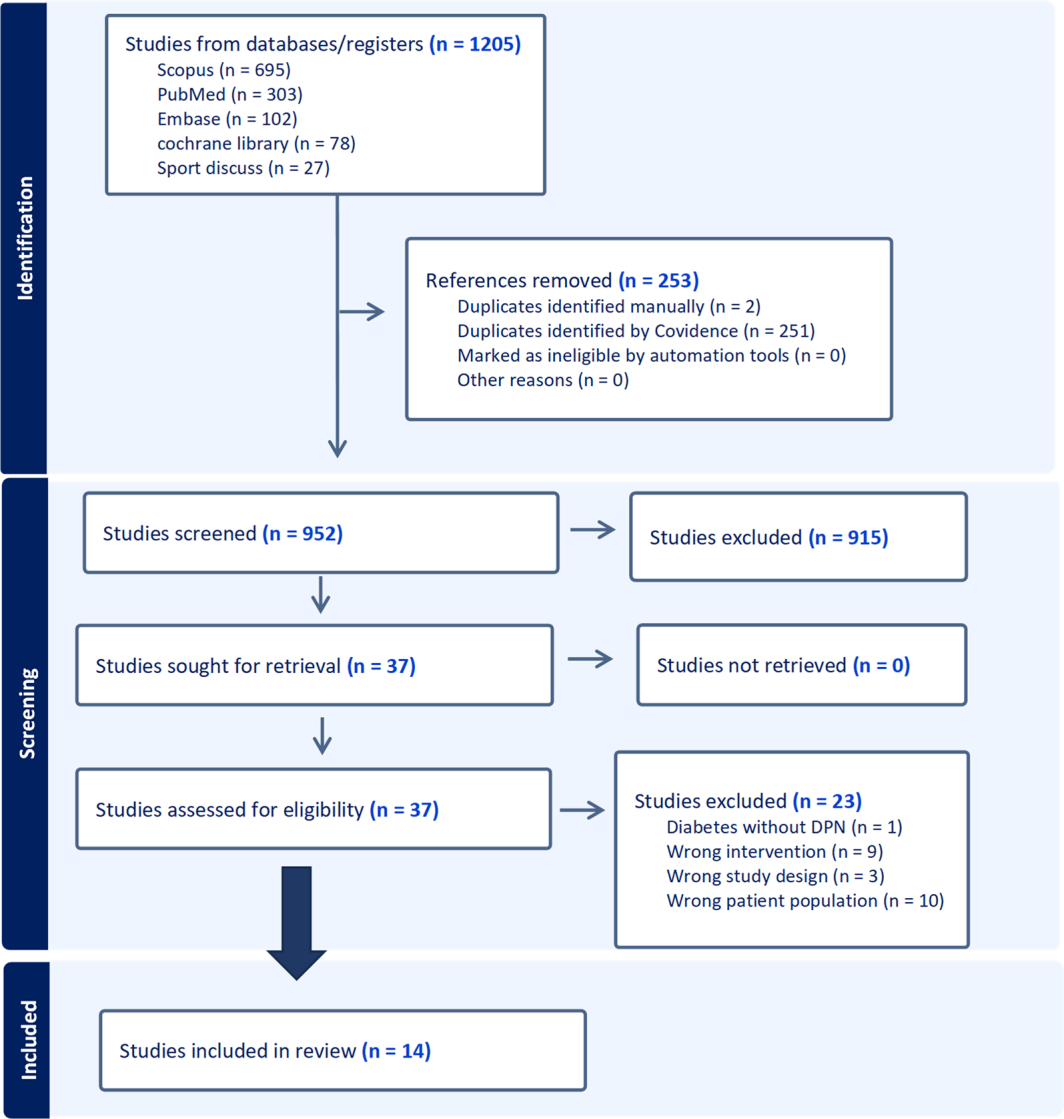
Flow diagram of selected studies

From the 1205 documents retrieved through the search, the titles and abstracts of 952 were reviewed, as the remainder were duplicates. After this initial screening, the full texts of 37 articles were downloaded and reviewed. Following the exclusion of 23 articles (Supplementary Material 2), a total of 14 systematic reviews were included in this umbrella review [27–40], six of which conducted meta-analyses [27, 28, 33, 35, 38, 39] (Fig. 1).

The characteristics of the included systematic reviews are shown in Table 1. The most recent search was conducted by Hernando-Garijo et al.[39] and various databases were used in the searches, with the most common being Pubmed (n = 17), Embase (n = 8), Cochrane Library (n = 10), Physiotherapy Evidence Database, PEDro (n = 8), Scopus (n = 9), and Web of Science (n = 8).

**Table 1:** Main characteristics of the included systematics reviews (n = 14)

Author, publication year	Period search	Study population	Intervention	Comparator	Outcomes	Databases	Type of studies	Quality appraisal tool (score range)
Akbari, 2022 [43]	Mar 1984–Feb 2020	Adults with DPN	Any exercise therapy	No treatment or other treatment	Static, dynamic, and functional balance	Medline, WoS, and Science Direct	RCT	PEDro scale (5–9)
de Oliveira-Lima, 2021 [44]	1966–Dec 2019	Adults with DPN	Exercise program to improve balance and strength of the lower extremities	Patients who did not participate in any kind of exercise program	Assessments of mobility, balance, and quality of life	Embase, Medline, LILACS, CENTRAL, Trip Medical Database, SCOPUS, Web of Science (WOS), Physiotherapy Evidence (PEDro) and ClinicalTrials.gov	RCT	Criteria described in the Cochrane Reviewers Handbook
Dixit, 2020 [45]	Jan 1995–Nov 2016	Diagnosis of T2DM > 5y with confirmed subclinical or clinical form of DPN	Task-oriented balance training, treadmill exercise, strengthening exercise, WBV, pilates, tai chi and yoga	Standard form of physical therapy care or conventional form of physical therapy	Static postural control	Medline, CINAHL, EMBASE, PROQUEST, Science Direct, Cochrane Library, Physiotherapy Evidence Database (PEDro) and Google Scholar	RCT	PEDro scale (5–8)
Gu & Dennis, 2017 [41]	2000–28 Sep 2014	Diagnosis of T2DM and DPN confirmed by an electrodiagnostic test, monofilament sensory test or vibration test	Lower limb strengthening exercises, balance exercises, walking programs, and Tai Chi	No treatment or other treatment	Balance, falls, lower limb muscle strength, 6MWT or TUG	Medline, Embase, CINHAL, PEDRO, and SCOPUS	Experimental and quasiexperimental studies and one case report	PEDro scale (3–8)
Gu, 2019 [51]	Inception–4 Dec 2017	Physician‐diagnosis of pre‐diabetes or T2DM with or without DPN	Any type of exercise training	No treatment or other treatment	Nerve function or nerve conduction parameters or neuropathy‐related questionnaires	Medline, CINAHL, AMED, PEDro,Cochrane Library, Embase, and Scopus	All types	PEDro scale (1–7)
Hernádez-Secorún, 2021 [48]	26 Nov 2020–20 Jan 2021	Individuals with DPN	Manual therapy and/or exercise;	Comparing with the control group and/or placebo and/or other conservative treatment	Balance, motor and sensory functions, and/or pain	Medline, WOS, Scopus, Cochrane, PEDro	RCT	PEDro scale and Cochrane’s Risk of Bias 2 (RoB 2)
Hernando-Garijo, 2023 [50]	Inception–Feb 24,2023	Patients diagnosed with DPN in the lower limbs	Any physical activity (e.g. aerobic, strengthening, sensorimotor or its combination)	Usual care, non-exercise conservative interventions, or no interventions	Neuropathic symptoms, signs, psychosocial aspects and/or physical function	Medline, WOS, PEDro and Cochrane Library	RCT	PEDro scale (4–8)
Lepesis, 2023 [38]	Inception–Apr 2022	People with DPN	Foot and ankle mobilisations and home stretches	Standard care	Ankle and big toe joint ROM, PPPs and improving balance	Medline, EBSCO, Cochrane Database of Systematic Reviews, Joanna Briggs Institute Database of Systematic Reviews and PROSPERO	RCT, QES, singles case studies and prospective cohort studies	CASP RCT Standard checklist (6–10);RoB-2 (2–6); CASP checklist for Cohort studies(3–59; ROBINS‐was used for non‐RCT studies
Matos, 2018 [47]	Inception–Jul 2017	Patients with a diagnosis of diabetes regardless of etiology, or a clinical diagnosis of DPN, polyneuropathy or DFU	Any form of supervised physical activity at a care center or at home	Daily-life physical activity and/or usual foot care education	Nerve conduction velocity; Neuropathic signs and symptoms, blood glucose	Medline; Cochrane Library; WOS and Scopus	CCT	PEDro scale (5–10)
Melese, 2020 [40]	Oct 2010–May 2020	Adults with DM, patients with DPN	Effectiveness of exercise: muscle strengthening training, circuit exercise training, range of motion exercise, balance training exercise, flexibility and stretching exercises, gait exercise training, and sensor-based interactive exercise training	Conventional therapy and/usual medical care/no intervention	Gait funtion, ROM, Tineti scale,	Medline, CINAHL, AMED, PEDro, ScienceDirect, Google Scholar, Cochrane Library	RCT	PEDro scale (6–8)
Mohamed & Jan, 2020 [42]	1990–2016	Older adults > = 55 years old; DM, DPN and propioception disfuntion	Propioceptive exercise to improve balance control	No treatment or other treatment	Balance Control, Proprioception, Psychological and Neuropathy	MedLine, Scopus, Web of Science (Thomson Reuters), CrossRef and Academic Search Complete PLUS (EBSCO))	RCT	Jadad scale (2–7)
Tatikola, 2022 [49]	Jan 2010–Dec 2021	Neuropathic pain in T2DM individuals; both male and female who are aged between 30 and 65 years	Exercise program for neuropathic pain in T2DM individuals	No treatment or other treatment	Intensity, frequency, mode, and duration of exercise program along with measures of MNSI, HbA1c, BMI, VAS, FBG, PPBG	Scopus, Web of Science, Medline, ScienceDirect, ProQuest	Single group studies, comparative studies, randomized controlled trials	Modified Downs and Black checklist High Methodological Quality Studies (n = 3) Moderate Methodological Quality Studies (n = 4)
Thurkal, 2021 [46]	Inception–Feb 2019	Patients suffering from DPN	exercise protocols on posture and balance in patients suffering from DPN	Standard care or nothing	Berg Balance Scale, TUGT and Balance System(posturography) for assessing balance and postural disturbances)	Medline, CTRI, PEDro, Cochrane database and Google Scholar	RCT	PEDro; Cochrane Collaboration tool
Van Netten, 2023 [39]	Inception–Mar 9, 2022	People at risk of foot ulceration (IWGDF 1–3)	Any physical activity specifically targeting any part of the foot, ankle or lower leg that is delivered in a predefined and structured programme with specified time, content and supervision, and with the aim of changing foot function	No treatment or other treatment	Ulceration or pre-ulcerative lesion, adverse events, peak plantar pressure, quality of life, ROM, DPN signs and symptoms, muscle strength and function	Medline, EMBASE Cochrane databases	RCT AND not randomiced	GRADE Y SIGN Risk of Bias Very low risk of bias (n = 5) Low risk of bias (n = 6) High risk of bias (n = 5)

*RCT*: Randomized Controlled Trial; *DPN*: Diabetic Peripheral Neuropathy; *PEDro*: Physiotherapy Evidence Database; *WOS*: Web of Science; *T2DM*:Type 2Diabetes Mellitus; *6MWT*: 6-min walk test; *TUGT*: Timed Up and Go test; *CINAHL*: Cumulative Index to Nursing and Allied Health Literature; *AMED*: Allied and Complementary Medicine Database; *WBV*: Whole-Body Vibration; *DM*: Diabetes Mellitus; *IWGDF*: International Working Group on the Diabetic Foot; *GRADE*: Grading of Recommendations Assessment, Development, and Evaluation; *SIGN*: Scottish Intercollegiate Guidelines Network; *ROM*: Range of Motion; *MNSI*:Michigan neuropathy screening instrument score; *HbA1c*: glycated haemoglobin levels; *BMI*: body mass index; *VAS*: visual analog scale; *FBG*: fasting blood glucose; *PPBG*: post prandial blood glucose; *CCT*: Controlled clinical trial; *QES*: Quasi-experimental study; *DFU*: Diabetic foot ulcer; *PPPs*: reducing plantar pressure points; *CTRI*:Clinical Trials Registry

Eight reviews [29, 31–35, 37, 39] included only randomized clinical trials (RCTs). The objectives of the reviews varied, focusing on patients with DPN, exploring any type of exercise (n = 5) or specific exercises (n = 9) such as balance training, walking programs, or Tai Chi, among others, compared with standard clinical practice or other interventions. The studies evaluate different outcomes, such as balance, muscle strength, nerve function, mobility, quality of life, falls, plantar pressure, DPN signs and symptoms, and blood glucose levels. The tools used to measure these parameters vary, ranging from scales, questionnaires, nerve conduction studies, clinical tests, and blood analyses, all of which differ among the various studies, leading to a significant heterogeneity of results. Regarding the study population, most reviews included studies on adult patients with DPN without specifically detailing the signs or symptoms present (n = 10) without age or type of diabetes filter, two reviews focused on a specific age range [31, 38] and the reviews by Gu et al. in 2019 [40], Van Netten et al. in 2023 [28] included studies on populations with diabetes that may or may not have DPN. However, for the present umbrella review, data were extracted only from studies conducted on patients with DPN.

The number of included studies, total sample size, location, and age and sex distribution of the study populations included in each review are presented in Table 2. The reviews included between six and 29 studies with a total N ranging from 298 to 1476 participants. Only eight reviews reported the location of the studies, 11 provided information on age, and four reviews provided information on sex distribution.

**Table 2:** Further characteristics of the included systematics reviews (n = 14)

Author, publication year	Number of included studies (Total sample size)	Number of excluded studies after review *	Location	Publication period	Age and sex distribution	Meta-analysis	Review author’s conclusion	Umbrella review authors’ assessment of conclusions
Akbari, 2022 [32]	12 (N = 580)	3	Not reported	1995–2020	From 49.73 ± 6.79 to 66.3 (10.6) and 34%−100% female	No	Balance and strengthening exercises are more effective on predominantly static balance indices	Various studies on balance and strength training interventions show several outcomes. Balance training with Biodex improved TUG, Berg Balance Scale, Biodex balance system, and fall risk indices. Short-term strength and balance training didn’t affect HRQoL but improved functional status and balance confidence over six months. Progressive sensorimotor and gait training enhanced proprioception and nerve conduction velocity. A program aimed at reducing blood glucose and HbA1c levels, and neuropathy TSSs also improved balance and quality of life dimensions. Physiotherapy interventions modestly optimized foot rollover dynamics and enhanced ankle function. Some interventions increased muscle strength and balance, and the EPN group saw improved static and dynamic balance. One training program didn’t improve balance or lower-extremity strength or affect the falling rate. Task-oriented balance training enhanced dynamic, anticipatory, and reactive balance
de Oliveira-Lima, 2021 [33]	8 (N = 457)	0	South Korea (n = 2), United States,Switzeland, Brazil, India (n = 2), United States & Qatar	2010–2015	Not reported	Yes	Exercise therapy provides short-term benefits in neuropathic symptoms, signs, and physical function in patients with DN is very low	A combination of gait, balance, and functional training improves balance, fear of falling, and quality of life, but not the risk of falls
Dixit, 2020 [34]	8 (365)	1	Not reported	2010–2016	Not reported	No	Exercise therapy provides short-term benefits in neuropathic symptoms, signs, and physical function in patients with DN is very low	Most of the included studies reported various forms of measurement protocols for static postural control. Despite the variations, task-specific balance training and whole-body vibration (WBV) appear to be the most effective interventions for improving balance in DPN. With moderate intensity, aerobic training on the treadmill showed an improvement in one variable of sway velocity
Gu & Dennis, 2017 [30]	10 (N = 422)	2	USA (n = 6), Italy, India, Switzerland, South Korea	2001–2014	58.9 (1.9) to 76 years	No	A targeted multicomponent training protocol, which includes functional strengthening exercises, walking programs, balance training or Tai Chi, can promote improvements in gait, balance and functional activity in people with DPN	Tai Chi, aerobic exercise, and weight-bearing progressive balance exercises improved balance, neuropathy scores, gait metrics, and step counts. Circuit training enhanced gait speed, balance, muscle strength, and joint mobility. FBT and TC improved different aspects of gait and balance. Multimodal manual treatment positively affected gait endurance and overall functionality. Overall, tailored exercise interventions provided significant benefits compared to usual care or less intensive exercises
Gu, 2019 [40]	12 (N = 298)	6	India (n = 2), USA (n = 4), South Korea, Taiwan, Iran	2002–2017	54 to 68 years	No	Preliminary evidence shows that moderate‐intensity aerobic exercise (undertaken for at least 150 min/wk) may positively influence nerve function, even among those with T2DM and DPN, with low risk of major adverse events. Based on the limited evidence to date, combining balance or Strength training exercise with aerobic exercise. did not show superior effect on nerve function to aerobic exercise alone. However, balance and strength exercises have been demonstrated to improve impaired balance associated with DPN	6 studies were excluded upon reviewing the individual studies. The interventions are based on Tai Chi, balance work with and without Biodex, aerobic exercise, and aerobic + strength exercise. Aerobic exercise shows good results in improving nerve function; however, these interventions also coincide with the longest intervention times and the highest weekly exercise frequency
Hernádez-Secorún, 2021 [37]	29 (N = 1476)	8	Not reported	2001–2020	Not reported	No	Exercise therapy provides short-term benefits in neuropathic symptoms, signs, and physical function in patients with DN is very low	8 articles did not meet inclusion criteria because they did not involve people with diabetes, interventions that were not exercises such as manual therapy, or that employed vibration without exercise. It mixes a wide variety of physical exercise interventions, making it difficult to draw a final conclusion. The outcomes are very varied, focusing more on measuring balance, and it provides a qualitative synthesis of the results. Overall, good results were observed after physical intervention, but the evidence to support this is low
Hernando-Garijo, 2023 [39]	11 (N = 517)	1	Brazil (n = 3), Czech Republic, Australia, Iran (n = 3), Saudi Arabia, Singapore, Canada	2014–2022	52 to 72 years, 50.1% were women and 49.9% men	Yes	The results of this systematic review and meta-analysis found very low quality of evidence suggesting that exercise therapy provide short-term benefits in neuropathic symptoms, signs, and physical function in patients with DN, but no effects were found in psychosocial aspects Physical activity and exercise are effective non-pharmacological interventions to improve diabetic foot-related outcomes	Overall quality of evidence is very low, with statistically significant improvements in symptoms, neuropathic signs, and physical function in favor of exercise therapy. No findings in psychosocial aspects. The Interventions were based on strength exercises, stretching and flexibility, and aerobic exercises, with no additional conclusions comparing one type of exercise with another
Lepesis, 2023 [27]	9 (N = 342)	2	Australia, Turkey, Brazil(n = 2), Egypt(n = 2), USA(N = 2), Belgium,	2002–2019	Age from 56,6(38–66) to 67,4(10.9); 9%−63%female	Yes	Exercises may increase ankle ROM and reduce forefoot PPPs	Methodological flaws, heterogeneity of study designs and lack of a gold standard of physical therapy intervention or treatment protocol meant that firm conclusions are not easy to reach. Future studies need to design their exercise programmes in partnership with PPI that are pragmatic and acceptable to participants and healthcare providers. The literature also needs to strive towards a universally recognised language when it comes to exercise prescription as the variability of terminology used generates poor evidence for the effectiveness of exercise in people with DPN. Longterm studies also need to be carried out with a shift of focus on patient reported outcome measures that are meaningful to patients
Matos, 2018 [36]	6 (N = 428)	1	Not reported	2006–2014	From 49 ± 15.5 years to 66.6 ± 10.4 years	No	This systematic review suggests physical activity and exercise as an efficient intervention to reduce the risk of diabetic foot. Although the variety of physical activity and exercise methods implemented in the trials, like aerobic exercise or combined modes of exercise, all have brought benefits in diabetic foot related outcomes	The sample size is very small for the intended objective. The exercise interventions include Tai Chi, gait training, treadmill, aerobic, and lower limb strength training (very different from each other). The main outcomes are MNSI, NCV, and the Semmes–Weinstein 10 mg monofilament. The interventions generally show good results in these aspects
Melese, 2020 [29]	9 (N = 370)	0	Switzerland, (n = 1), Netherlands(n = 1), Brazil(n = 1), Japan(n = 1), Italy(n = 1), USA,(n = 2), Egypt (n = 2)	2010–2019	65.2 (12.8)to 73 (10)	No	Exercise therapy is found to improve gait function of patients with DPN. Specific exercise training programs, including range of motion, muscle strengthening, circuit training, stretching exercise, gait, and balance exercises can improve gait of diabetic patients with DPN	The interventions were different from each other, so similar interventions cannot be compared. Additionally, the gait functions of the participants were evaluated using different outcome measures, making it difficult to generalize which parameter primarily improves. Some report improvements in walking time, others in the percentage increase in stride length. Overall, it is observed that these multicomponent exercise interventions, such as strength exercises, range of motion exercises, balance exercises, flexibility and stretching exercises, circuit training, and gait training, improve gait function in people suffering from diabetic peripheral neuropathy compared to control groups
Mohamed & Jan, 2020 [31]	9 (N = 464)	3	Not reported	2010–2015	From 57.7 ± 6.4 to 76.31 ± 4.78	No	This systematic review shows that proprioceptive exercise is a vital component of any training performed for older adults with DM in order to get a fast improvement in balance control and a decrease in fall risk. Also, this systematic review recommends the early performance of the proprioceptive exercise in any balance training to encourage those people for more participation in the exercise program	Various balance and proprioception exercise programs seem to improve stability in older adults with diabetes Exercise therapy provides short-term benefits in neuropathic symptoms, signs, and physical function in patients with DN is very low
Tatikola, 2022 [38]	9 (N = 409)	0	India (n = 2), Iran (n = 3), USA (n = 3), Australia (n = 1)	2012–2019	222 females; 187 males	Yes	In people at risk of foot ulceration, a foot–ankle exercise programme of 8–12 weeks duration may not prevent or cause diabetes-related foot ulceration. However, such a programme likely improves the ankle joint and first metatarsalphalangeal joint range of motion and neuropathy signs and symptoms. Further research is needed to strengthen the evidence base, and should also focus on the effects of specific components of foot–ankle exercise programs	There is a great heterogeneity in the exercise programs and in the measurement tools, clear conclusions cannot be drawn as some results are contradictory
Thurkal, 2021 [35]	16(n = 841)	3	Pakistan, Thailand, Malaysia, Egypt (n = 2), Iran (n = 2), Korea (n = 3), USA, Saudi Arabia, Spain, China, Switzerland, India and Australia	2003–2018	Not reported	Yes	Exercise protocols can result in significant improvement of balance in patients of DPN	Evaluation of exercise on posture and balance, 3 do not meet inclusion criteria, mixes patients with and without neuropathy, extracted by outcomes measure, Berg Balance Scale, TUGT and Biodex Balance System. The interventions are based on treadmill, whole body vibration training, Biodex, balance, gait training, Tai Chi ball, Swiss ball training, and Frenkel training. These exercise protocols are beneficial in improving balance and posture, measured in terms of changes in TUGT scores, Berg Balance Scale, and postural balance
Van Netten, 2023 [28]	29 (N = 977)	4	Not reported	2002–2022	Not reported	Yes	In people at risk of foot ulceration, a foot–ankle exercise program of 8–12 weeks may not prevent or cause diabetes-related foot ulceration. However, such a program likely improves the ankle joint and first metatarsophalangeal joint range of motion, as well as DPN signs and symptoms	Some of the studies do not include patients with DPN, and four are excluded for this reason. It provides a general analysis of exercise studies on the foot and addresses safety and improvement in terms of plantar pressures, mobility, and improvement in DPN

*HRQoL*: Health-Related Quality of Life; *TSS*: Total Symptom Score; *HbA1c*: Hemoglobin A1c; *TUGT*: Timed Up and Go test; *DPN*: Diabetic Peripheral Neuropathy; *T2DM*: Type 2Diabetes Mellitus; *6MWT*: 6-min walk test; *PPPs*: reducing plantar pressure points; *ROM*: Range of Motion; *WBV*: whole-body vibration; *FBT*: Functional Balance Training; *TC*: Tai Chi. * After reviewing each of the individual studies included in each of the reviews in our Umbrella Review, those that did not meet our inclusion criteria were excluded

The total number of individual studies included in the systematic reviews was 180. After removing duplicates (n = 76) and studies that did not meet the eligibility criteria (n = 34) for this umbrella review, either because the included systematic review had broader criteria or due to selection errors by the authors, the total number of individual studies that met the eligibility criteria for this umbrella review was 70. Supplementary Material 3 details the intervention studied and the results of each individual study, and Supplementary Material 4 details the total number of studies included in each review and specifies which of them were excluded after verifying that they did not fit the population included in this umbrella review. Results are based on data extracted from 70 eligible individual studies included in the systematic reviews.

### Type of Exercise Training

The exercises studied varied in modality, duration, and frequency, with nearly every intervention being different. To synthesize and structure the results, they have been categorized into seven categories:

- Balance: Includes interventions aimed at improving balance capacity, postural stability, and proprioception through specific exercises with and without the use of technological tools. One of the most commonly used tools is the Biodex® Balance System (BBS) [41, 42] (used in exercises by Eftekhar-Sadat B [43], 2015, Ravand 2021[44], Salsabili H, 2011 [45]) This advanced balance platform is used to assess and improve postural stability and balance control, providing real-time visual feedback. Exercises include lateral and anteroposterior weight shifts, limits of stability, and unstable surface training, all designed to improve lower limb stability. This system helps strengthen stabilizing muscles, improve postural control, and reduce the risk of falls. Additionally, the Computerized Evaluation and Re-education Biofeedback (CERB) technique has been employed to evaluate and enhance postural control and balance stability, combining precise balance assessment with real-time feedback to help individuals correct and improve their balance skills. Vibration platforms, specifically Whole-Body Vibration (WBV) [46], have also been used to enhance balance and muscle strength, but only the interventions which included exercise (active movement) were considered in this umbrella review.
- Aerobic Exercise: Interventions based on moderate to intense supervised aerobic exercise, such as walking or treadmill running, cycling, elliptical exercises, and combined aerobic and resistance HIIT(C-HIIT) and combined aerobic and resistance MICT (C-MICT) [47].
- Stretching and Flexibility: Includes static and dynamic stretches (controlled movements such as arm circles, leg swings, and trunk twists) to improve flexibility, specific stretches aimed at enhancing joint mobility in the foot and ankle, and general stretches to improve overall body mobility. The terms stretching and flexibility are used because the differences between flexibility exercises and stretching exercises are subtle but important, particularly in their focus and purpose. Flexibility exercises aim to improve the range of motion of a joint or group of joints, while stretching exercises specifically focus on lengthening muscles and tendons, which can be static or dynamic and are typically performed to enhance muscle elasticity, increase muscle control, and reduce injury risk.
- Strength: Includes exercises that work on muscle strength through various methods, including group training, home exercises, weight lifting, whole-body vibration, and specific regimens to strengthen the ankle and foot muscles. Some interventions refer to strength training exercises, which often involve isometric exercises, such as planks or sustained muscle contractions, as well as dynamic exercises, like squats, core work, weight lifting, and individualized training circuits focused on developing overall muscle strength. Strength and resistance exercises are used as synonyms in diabetes research and exercise in diabetes. The effects of strength/resistance exercises on the muscular system are modulated by various aspects such as intensity, volume, frequency, etc. Depending on these variables, several muscle improvements can be promoted, such as strength, power, muscle mass, and muscular endurance, as well as improved glycemic control and insulin sensitivity.
- Gait: Includes sensorimotor training to improve gait; exercises designed to enhance gait coordination and efficiency; Biofeedback Training, which involves 10-step walking sequences followed by subjective performance estimation and objective visual feedback using the PEDAR® System, providing a graphical representation of plantar pressure during gait; and gait practice with visual (computer) and verbal (professional) feedback, focusing on initiating leg swing from the hip rather than pushing with the foot. This category does not refer to walking; when walking is used as training, it is included in the aerobic category.
- Combined: Includes interventions that combine any exercise described in the previous categories. Tai Chi is also included in this category, as it encompasses aerobic capacity, strength, flexibility, and balance training
- Miscellaneous: Encompasses various interventions that do not easily fit into the other established categories. These interventions include exercise programs and activities that combine multiple approaches or are unique in their design. This includes Buerger exercises, Swiss Ball training, Frenkel training, aquatic exercise, active foot exercises in dorsiflexion/plantarflexion directions, sensorimotor training to improve protective sensation, or general exercise without detailed specifications.

In most studies, the prescribed exercise is added to the standard therapy and compared with routine clinical practice and usual patient care. Less frequently, it is compared with other types of exercise, educational interventions, passive physiotherapy or manual techniques, or sedentary behavior. Only one study [48] includes more than one comparator group (Supplementary Material 3).

### Duration and Frequency of Exercise

The duration of the interventions ranged from 3 to 24 weeks, with a frequency varying from 2 to 5–7 times per week, predominantly consisting of one-hour sessions three times per week. The study by York RM in 2009 [49] had a shorter duration, conducted over just 2 days, and the study by Rodríguez in 2013 [50] spanned 10 days. These are the only studies with such a brief intervention period, as both investigated changes in plantar pressures by modifying gait dynamics. In contrast, the study by Smith AG, et al. [51] was the longest, conducted over a period of 1 year (mo).

### Age Range and Sex Distribution

The sample size of the individual studies was less than 100, except for the study by Venkataraman K, 2019, which included 143 participants. The mean age reported in the reviews ranged from 50 to 70 years. As for sex distribution, which was not consistently detailed in most systematic reviews, data were retrieved directly from the individual studies. Most studies had a good balance, except for the studies by Nadi, et al. [52], and Hedayati A, et al. [53], which only selected women, and the studies by Gholami F, et al. [54], and Gholami F, et al. [55], which only selected men*.*

### Outcome Measures and Assessment Tools

Table 3 reports the outcome measures evaluated in the studies included in the systematic reviews considered in this umbrella review and the tools used for their assessment. Static balance was commonly measured using the Biodex Balance System platform (BBS) (n = 9) and the single leg stance (SLS) (Unipedal stance test—open and closed eyes) (n = 8). Dynamic balance was assessed through the Berg Balance Scale (BBS) (n = 11) and the Timed Up and Go (TUG) test (n = 13).

**Table 3:** Study outcome variables and assessment tools

Outcome	Tool	Frequency
Static balance	Balance platform	4
	Single leg stance (SLS) (Unipedal stance test- open and close eyes)	8
	Postural sway with eyes open	2
	Postural sway with eyes closed	2
	Body sway distance test	1
	Biodex balance system platform	9
	Metitur good balance system	1
Dynamic/functional balance	Berg balance scale (BBS)	11
	Timed Up and Go (TUG) test	13
	Functional reach test (FRT)	2
	Five-times-sit-to-stand (FTSTS)	2
	Star excursion balance test (SEBT)	2
	Performance-oriented mobility assessment (POMA) scale (Tinetti test)	1
	Confidence (ABS Scale)	3
Fall risk	Fall risk index	2
	Fall efficacy scale international (FES-I)	3
Health-related quality of life (HRQoL)	Short-form health survey (SF-12)	2
	SF-36v2	3
	EQ-5D-5L index score	1
	Function index disability scale (FIDS)	1
	Hospital anxiety and depression scale (HADS)	1
	Beck Depression Inventory-II	1
	Quality of life (QoL)	3
Proprioception	Pedalo®-sensamove balance test pro with miniboard	2
	Surface electromyography	2
Nerve conduction and function	Intraepidermal nerve fiber density through a skin biopsy	3
	Nerve conduction velocity (NCV)	5
	Valk neuropathy severity scores	2
	Nerve function measures	2
	Electrophysiological evaluation (Peroneal and sural sensory motor nerves)	2
	Surface electromyography	2
Glucose control	Postprandial blood glucose	1
	Fasting blood glucose (FBG)	6
	Glycated hemoglobin (HbA1c)	9
Neuropathy score	Total symptom score (TSS)	3
	Semmes–Weinstein monofilament examination (SWME)	5
	Michigan neuropathy screening instrument (MNSI)	9
	Michigan diabetic neuropathy score (MDNS)	6
	Vibration perception threshold (VPT)	3
Strength	Right-ankle dynamometry	4
	Back-leg-chest dynamometer	2
	Hand-held dynamometer	2
	Micro FET2 (Isometric strength measurement)	1
Gait	Habitual walking speed (m/s)	1
	6-min walk test (6MWT)	5
	10-m walking test	1
	Performance-oriented mobility assessment (POMA) scale (Tinetti test)	2
	Daily step counts (StepWatch Activity Monitor)	1
	Walking velocity, cadence, step time, double support time	5
	Physical activity level and self-selected/fast-gait speeds	2
	Highest pressure at baseline PEDAR (®)	3
Joint mobility	Manual goniometer (for ankle, foot and first metatarsophalangeal joint ROM)	8
Foot function and PSS	Biomechanical modifications during gait	2
	Foot and ankle ability measure (FAAM)	1
	Peak plantar pressure	8
Pain	Visual Analog scale (VAS)	7
	Neuropathic pain symptom inventory (NPSI)	2
Physical function and mobility	30-s chair stand test (30 s-CST)	1
	5 Times sit-to-stand test (5STS)	3
	Functional reach test (FRT)	3
	Assessment of lower extremity strength and endurance	1
Safety	Ulcer incidence	1
	Adverse events	1
Weight variation	Body mass index (BMI)	2
Peripheral arterial disease	Ankle-brachial index (ABI)	2

Fall risk was typically measured by the Fall Risk Index (n = 2) and the Fall Efficacy Scale International (FES-I) (n = 3). Quality of life was assessed using various questionnaires and was a less commonly reported area, primarily evaluated with the Short-form Health Survey (SF-12) and SF-36v2. Proprioception was evaluated using the Pedalo®-Sensamove Balance Test Pro system. Nerve conduction and its variations were assessed using a wide range of tools, with the most commonly reported being Nerve Conduction Velocity (NCV). To evaluate changes in DPN improvement, the Michigan Neuropathy Screening Instrument (MNSI) (n = 9) and the Michigan Diabetic Neuropathy Score (MDNS) (n = 6) were used. For assessing improvements in glycemic control, the main tool used was the measurement of Glycated Hemoglobin (HbA1c).

Strength was evaluated using a dynamometer adapted to each anatomical region, and joint mobility was assessed with a manual goniometer, varying the area to be examined. Changes in gait were typically assessed by analyzing walking velocity, cadence, step time, and double support time (n = 5), while functional capacity was measured using the 6-min walk test (6MWT) (n = 5). The safety of exercise in participants was recorded by noting adverse events or the incidence of ulcers, although this was reported by only a few studies. One study evaluated vascular status using the Ankle-Brachial Index (ABI) and weight variation using the Body Mass Index (BMI).

### Effect of Exercise Training

Table 4 summarizes the impact of different types of exercise on each of the most commonly evaluated outcomes in the studies included in the reviews. This table has been generated with the results extracted from the individual studies (see supplementary material 3). Significant improvement is represented by a plus sign (+) when the improvement is significant overall, by a ‘ + *’ when it is significant in some parameter items but not in all, and by a ‘–’ when there are no statistically significant differences.

**Table 4:**
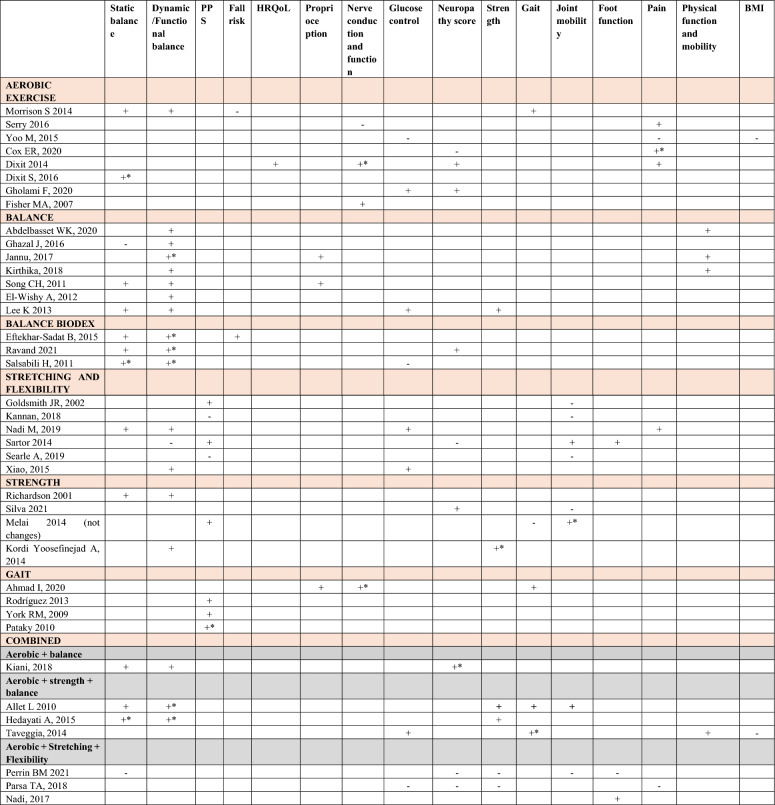

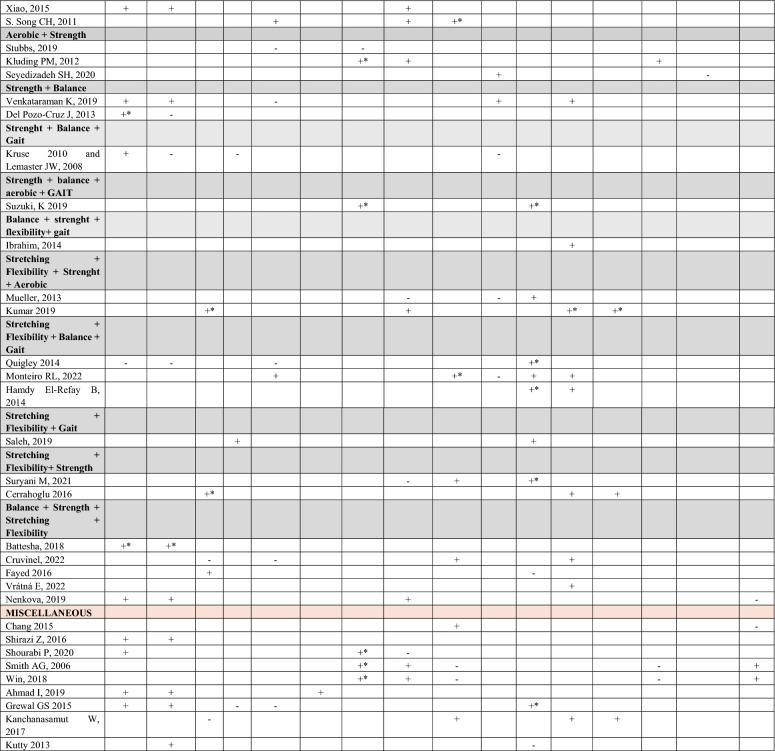
Improvement of outcomes by exercise category

Uppercase letter and bold type: Main category; Lowercase letter and bold type: Subcategories; PPS: reducing plantar pressure points; HRQoL: Health-Related Quality of Life; *Symbols:* + *Significant improvement;* + * Improvement significant in only some items of the parameter; –No statistically significant differences. The full references are included in Supplementary Material 4

The evidence indicates that aerobic exercise interventions improve static balance, dynamic balance, nerve conduction, glycemic control, joint mobility, neuropathy score and neuropathic pain. The most reported improvements are in neuropathy score, balance, pain, and glycemic control, with the latter also showing improvements when combined with other types of training such as strength training. In contrast, interventions focused solely on “balance” or “stretching and flexibility” showed good results in static and dynamic balance, but few studies observed improvements in reducing fall risk or proprioception, glycemic control, neuropathy score, strength, neuropathic pain, or physical function. Strength-based exercise interventions showed improvements in static and dynamic balance, neuropathic signs and symptoms, strength, and foot functionality in dynamic activities.

Regarding “gait” training as a standalone exercise, improvements were observed in reducing plantar pressures, which was the most notable outcome, followed by improvements in nerve conduction and general gait. However, gait stability and functionality improvements were more evident in interventions that included combinations of aerobic + strength + balance. The combination of aerobic + balance reported benefits in dynamic balance and pain. Aerobic + stretching + flexibility showed improvements in balance, neuropathic signs and symptoms, and glycemic control.

For aerobic + strength, improvements were observed in fall risk, nerve conduction velocity, glycemic control, strength, and pain reduction. Strength + balance reported improvements in static and dynamic balance, joint mobility and strength. The outcomes with the least improvement were joint mobility and BMI.

The combination of strength + balance + gait, with or without aerobic, only showed benefits in gait improvement. Similarly, the combination of stretching + flexibility + balance + gait and stretching + flexibility + balance or gait also showed improvements only in gait. However, combining balance + stretching + flexibility + strength reported advantages in dynamic balance, fall risk reduction, decreases in peak plantar pressures, improvements in neuropathy scores, joint mobility, foot function, and consequently, gait.

The miscellaneous category includes various types of exercises that improve different conditional capacities, such as Buerger exercises [56], Swiss Ball training, Frenkel training [57] (coordination), aquatic exercise, active foot exercises in the dorsiflexion/plantarflexion direction of the ankle, sensorimotor training to enhance protective sensation, or general exercise without detailed specification. Therefore, each intervention should be interpreted individually. The category of improvement in vascular function was not included in the table as it was a less studied variable. However, the studies by Chang, et al. [56], Gholami F, et al. [54], and Gholami F, et al. [55] reported improvements in this outcome.

Table 5 details the results of the meta-analyses conducted in the six reviews that included quantitative analysis. The results indicate that exercise training interventions can significantly improve glucose levels (fasting glucose and HbA1c) (n = 1) [38], neuropathic symptoms (n = 3) [28, 38, 39], physical function (n = 1) [39], balance (n = 2) [33, 35], postural sway with eyes closed, reduce fear of falling, and significantly increase the range of motion (n = 1) [33]. However, exercise training did not produce significant changes in BMI (n = 1) [38], the risk of ulcer incidence (n = 1) [28], the incidence of adverse events (n = 1) [28], weight-bearing activity (n = 1) [28], quality of life (n = 1) [28], or forefoot pressure points (n = 1) [27, 28]. For the remaining evaluated outcomes, the results were inconclusive due to the significant and high heterogeneity of the studies.

**Table 5:** Results of the included meta-analyses (n = 6)

Author, publication year	Intervention	Outcome variable	Number of RCTs (total sample size)	Global effect, 95%CI, p-value	Heterogeneity
Van Netten, 2023 [28]	Any physical activity specifically targeting any part of the foot, ankle or lower leg that is delivered in a predefined and structured programme with specified time, content and supervision, and with the aim of changing foot function	Ulcer incidence	5 (N = 274)	RR = 0.63; 95% CI 0.28 to 1.42; *p* = 0.26	I^2^ = 41%, p = 0.15
		Adverse events	6 (N = 435)	RR = 1.04; 95% CI 0.65 to 0.67; *p* = 0.87	I^2^ = 0%, *p* = 0.76
		Barefoot peak plantar pressure at the medial forefoot	5 (N = 116)	MD = −6.28; 95% CI − 69.90 to 57.34; *p* = 0.85	I^2^ = 81%, *p* = 0.006^a^
		Ankle range of motion	5 (N = 376)	MD = 1.49; 95% CI − 0.28 to 3.26 *p* = 0.10	I^2^ = 62%, *p* = 0.03^a^
		Neuropathy signs and symptoms (questionnaire score)	4 (N = 204)	MD = −1.42; 95% CI −2.95 to 0.12; *p* = 0.07	I^2^ = 89%, *p* = 0.00001^a^
		Weight-bearing activity	3 (N = 176)	MD = 130.96; 95% CI − 492.00 to 753.91; *p* = 0.68	I^2^ = 0%, *p* = 0.40
		QoL	2	MD = 0.02; 95% CI − 0.01 to 0.06; *p* = 0.175)	Not reported
Tatikola, 2022 [38]	Exercise program for neuropathic pain in T2DM individuals	BMI	4 (N = 137)	MD = 0.75; 95% CI − 0.89 to 2.39; *p* = 0.37	I^2^ = 0%, *p* = 0.87
		FBG	3 (N = 120)	MD = − 14.05; 95% CI − 26.60 to − 1.51; *p* = 0.03	I^2^ = 53%, *p* = 0.12
		Post pandrial blood glucose	3 (N = 119)	MD = − 24.44; 95% CI − 63.80 to 14.92; *p* = 0.22	I^2^ = 80%, p = 0.006^a^
		HbA1c	5 (N = 202)	MD = − 0.41; 95% CI − 0.69 to 0.12; p = 0.005	I^2^ = 36%, p = 0.18
		Neuropathic pain (MNSI)	3 (N = 114)	MD = − 2.92; 95% CI − 4.59 to 1.24; *p* = 0.0006	I^2^ = 88%, *p* = 0.0002^a^
		Neuropathicpain (VAS)	2 (N = 88)	MD = − 1.62; 95% CI − 6.35 to 3.12; p = 0.50	I^2^ = 91%, *p* = 0.0009^a^
Hernando-Garijo, 2023 [39]	Exercise- based interventions, defined as any physical activity (e.g. aerobic, strengthening, sensorimotor, or its combination)	Neuropatic symptoms	6 (N = 231)	MD = − 1.05; 95% CI − 1.90 to − 0.2.20; *p* = 0.02	I^2^ = 72%, *p* = 0.003^a^
		Neuropathic signs	6	MD = − 0.66; 95% CI − 1.00 to − 0,32; *p* = 0.0001	I^2^ = 43%, *p* = 0.14
		Psychosocial aspects (anxiety, depression)	2	MD = − 0.37; 95% CI − 0.92 to 0.18; *p* = 0,19	I^2^ = 66%, *p* = 0.03^a^
		Physical function for exercise therapy	4	MD = − 0.45; 95% CI − 0.66 to—0.24; *p* < 0,0001	I^2^ = 37%, *p* = 0.18
Lepesis, 2023 [27]	Foot and ankle mobilisations and home stretches	ROM	3 (N = 153)	MD = 1.76; 95% CI 0.78, 2.74; *p* = 0	I^2^ = 0%, *p* = 0.827
		Reducing PPPs	3 (N = 206)	MD = − 23.34; 95% CI − 59.80 to13.13; *p* = 0.21	I^2^ = 51%, *p* = 0.164
Thurkal, 2021 [35]	Exercises on posture and balance	Balance (TUGT scores)	10 (N = 458)	MD = 1.42; 95% CI 0.75 to 2.09; *p* < 0.0001	I^2^ = 84%, *p* < 0.00001^a^
		Balance (BBS)	9 (N = 361)	MD = − 2.25, 95% CI − 3.08 to − 1.42; *p* = 0.01	I^2^ = 62%, *p* < 0.00001^a^
		Postural sway with eyes open	4 (N = 172)	MD = − 9.88; 95% CI − 17.68 to − 2.08; *p* = 0.01	I^2^ = 100%, *p* < 0.00001^a^
		Postural sway with eyes closed	2 (N = 65)	MD = 0.85; 95% CI 0.33 to 1.37; *p* = 0.001	I^2^ = 0%, *p* = 0.001
de Oliveira-Lima, 2021 [33]	Exercise program to improve balance and strength of the lower extremities	Fear of falling (FES)	3 (N = 185)	MD = − 2.42; 95% CI − 4.70 to 0.15; *p* = 0.02	I^2^ = 1%, *p* = 0.36
		Balance (left leg -open eyes)	3 (N = 153)	MD = 3.70; 95% CI 0.64 to 6.76]; *p* = 0.02	I^2^ = 0%, *p* = 0.81
		Balance (left leg -closed eyes)	3 (N = 153)	MD = 1.07; 95% CI 0.34 to 1.79; *p* = 0.004	I^2^ = 0%, *p* = 0.78

^a^ The Global effect is not representative (inconclusive) due to the high heterogeneity

*PPPs*: peak plantar pressures; *TUGT* scores: Timed Up and Go Test scores; *ROM*: Range of Motion; *BBS*: Berg Balance Scale; *FES*: Fear of falling; *MNSI*: Michigan Neuropathy Screening Instrument; *QoL*: Quality of Life*; BMI*: Body Mass Index; *FBG*: Fasting Blood Glucose; *HbA1c*: Hemoglobin A1c; *VAS*: Visual Analog Scale; *MD*: mean differences; *ci*: Confidence Interval

### Methodological Quality of Included Reviews

Table 6 shows the results of the quality assessment of the included systematic reviews. All systematic reviews had more than one critical weakness, except for those by Oliveira et al. and Gu et al. [32, 35], which presented only one critical weakness along with other non-critical weaknesses. In general, items 4, 7, and 10, which may be related to reporting errors, were the least fulfilled by the reviews.

**Table 6:**
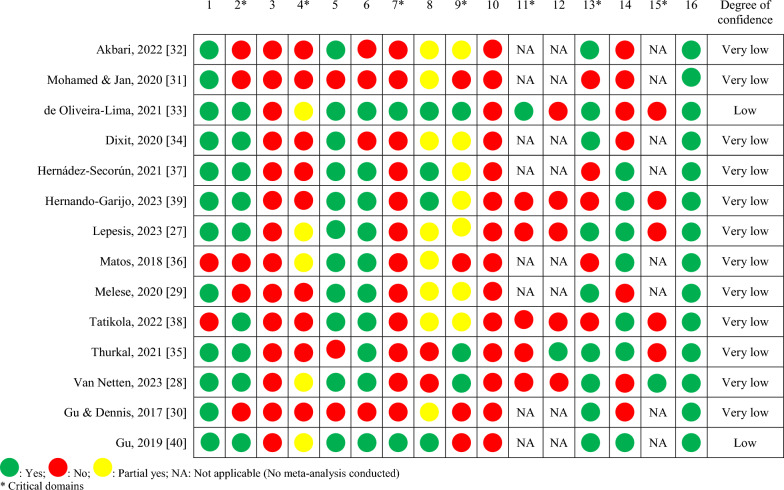
Quality assessment of the included systematic reviews using the AMSTAR-2 tool (n = 14)

## Discussion

This umbrella review synthesizes the accumulated evidence on the impact of exercise training interventions in patients with DPN. The reviews indicate that the studies conducted to date investigate very different exercise training interventions, focusing either on improving a single conditional capacity or several. To evaluate the benefit of exercise in patients with DPN, authors choose to assess various health outcomes such as static or dynamic balance, fall risk, proprioception, quality of life, neuropathic signs and symptoms, neuropathic pain, changes in nerve conduction, strength, gait changes, improved joint mobility, changes in plantar pressures, variations in glucose levels, incidence of adverse events like ulcer formation, or changes in BMI. They also use different tools to measure each of these outcomes.

The variability among the studies generally prevents the authors of the reviews from combining and analyzing the results of multiple independent studies through meta-analysis, and those that do find high heterogeneity for most outcomes. A total of nine meta-analyses included in five systematic reviews provide significant differences in the evaluated outcomes in favor of exercise training interventions, with low heterogeneity and offering the most robust conclusions: aerobic exercise, with or without strength training, over a duration of 8 to 16 weeks, can improve fasting blood glucose and HbA1c levels; exercise training reduces neuropathic symptoms and improves physical function; foot and ankle mobilizations and home stretching exercises increase range of motion; postural and balance exercises produce slight changes in postural sway with eyes closed, improve balance, and reduce fear of falling. Targeted interventions generally showed better results than non-targeted ones.

Although six of the included reviews performed meta-analyses, these focused on specific outcomes and well-defined interventions with low variability. In our umbrella review, the studies varied widely in terms of exercise types, duration, frequency, population characteristics, and outcome measures. This high level of heterogeneity made it inappropriate to combine results statistically. For this reason, we chose to present a structured qualitative synthesis instead of performing a new meta-analysis.

In this context, the recommendations proposed by the American Diabetes Association (ADA) [58], emphasize the importance of various types of exercises that people with diabetes should perform including aerobic exercise (e.g., walking, cycling, and swimming), strength training exercise (using machines, free weights, resistance bands, and/or body weight), and flexibility and balance exercises (stretching: static, dynamic, and other types of stretching; yoga; balance: practicing standing on one leg, exercises using balance equipment, lower-body and core exercises, tai chi); however, recommendations for individuals with DPN are very scarce. Although the scientific literature includes numerous systematic reviews exploring the impact of exercise training interventions in patients with DPN, the variability within and between these reviews highlights the need to aggregate them in the present umbrella Review. This approach provides an updated and synthesized evidence based on the benefits of exercise training for patients with DPN.

Most systematic reviews on exercise training in patients with DPN focus on evaluating the impact of various exercise interventions on functional stability [27, 30–35, 37]. This focus is understandable given that functional stability is crucial for reducing the risk of falls and improving mobility in this population [59]. However, it is equally important to consider that some reviews have focused on neuropathic status, addressing aspects such as neuropathic pain and nerve function. More sporadically, other reviews have explored the impact of exercise training on diabetes control indicators, psychological aspects, quality of life, incidence of ulcerations, and adverse events. These additional aspects are fundamental for a comprehensive understanding of the benefits of exercise training, as the successful management of DPN requires a multidimensional approach that not only improves physical function but also overall well-being and the prevention of complications [60]. Of the 14 systematic reviews analyzed, some focused primarily on aerobic exercise, such as those by Yu Gu et al. (2018), Matos et al. (2018), and Pallavi Tatikola et al. (2022), which evaluated its impact on nerve function and neuropathic pain. Regarding static balance, Dixit et al. (2020) and Lepesis et al. (2023) stood out for investigating postural control in quiet standing. Other studies, such as those by Mohamed et al. (2019) and Thukral et al. (2020), focused on dynamic balance, addressing stability during movement through interventions like Tai Chi and proprioceptive training. Several authors, including Akbari et al. (2022), De Oliveira Lima et al. (2021), and Yu Gu et al. (2017), assessed both static and dynamic balance, adopting a more functional perspective. In contrast, Melese et al. (2020) and Van Netten et al. (2023) prioritized the analysis of strength training programs, while studies such as those by Hernando-Garijo et al. (2023) and Hernández-Secorún et al. (2021) implemented multimodal interventions, integrating strength, balance, and aerobic exercise without a clearly predominant component. Regular exercise is an evidence-based recommendation for non-pharmacological therapy in patients with T2DM and coronary heart disease, with a focus on aerobic exercise due to its benefits on insulin sensitivity and vascular function [61–63]. However, strength training exercise has been shown to be more effective than aerobic exercise for improving glycemic control and body composition in individuals with normal-weight T2DM [64]. In fact, the combination of aerobic and strength training has been identified as the most effective for reducing HbA1c and improving other metabolic parameters, surpassing approaches that use only one type of exercise [58, 63, 65].

In patients with type 1 diabetes, recent studies have highlighted the importance of strength training for long-term glycemic control. According to Reddy et al. [66], strength training not only increases the time patients maintain normal glucose levels after exercise but also reduces hyperglycemia and the need for insulin in the 24 h following exercise, compared to aerobic exercise [66]. Additionally, Yardley et al. [67] found that performing strength training exercises before aerobic exercise improves glycemic stability and reduces the duration and severity of post-exercise hypoglycemia in people with type 1 diabetes [67]. Current guidelines recommend a minimum of 150 min of moderate to intense exercise per week, including at least two or three days of strength training, as part of a comprehensive approach to diabetes management [68].

Moreover, balance exercises are frequently investigated for their effectiveness in reducing fall risk and improving functional stability. DPN can cause altered sensitivity, pain, and muscle weakness that can lead to deformities such as hammer or claw toes, which in turn generate elevated plantar pressure due to the instability of the metatarsophalangeal joint, compromising balance and increasing fall risk [69]. Often, studies combine aerobic exercise with strength training, balance exercises, and stretching, as these combinations address multiple physical conditioning capacities and offer comprehensive benefits. Increasing muscle mass and strength can help compensate for muscle weakness caused by DPN [70], and joint range of motion through stretching can reduce stiffness, relieve muscle tension, and alleviate pain, which can be common in DPN [71]. Additionally, research has been conducted to analyze the effectiveness of gait training in reducing plantar pressures and improving nerve conduction.

This umbrella review included outcomes such as neuropathic signs and symptoms, which provide information on the progression of neuropathy; nerve conduction, which determines the degree of nerve damage; and gait metrics, which offer insights into the patient’s functional capacity and how neuropathy affects their mobility, have been evaluated with similar frequency, highlighting the importance of these factors in neuromuscular function and quality of life [72]. However, it is noteworthy that crucial variables such as fall risk, ulceration incidence, and adverse events are evaluated in very few studies, representing a significant area of opportunity for future research. Assessing the safety of these patients during exercise interventions is fundamental to ensuring that the implemented programs are not only effective but also safe.

As part of our secondary objective, we also analyzed the most commonly used assessment tools across the included reviews. For static balance, the most frequently reported measures were the Biodex Balance System and the Single Leg Stance test, commonly used to assess postural control in quiet standing. Dynamic balance was often evaluated using the Timed Up and Go test and the Berg Balance Scale, which are standard tools for assessing mobility and stability during movement. Neuropathy status was typically assessed using clinical instruments such as the Michigan Neuropathy Screening Instrument and the Michigan Diabetic Neuropathy Score, while nerve conduction studies frequently employed Nerve Conduction Velocity tests to objectively quantify peripheral nerve function. Glycemic control was most commonly monitored through HbA1c levels, and gait-related outcomes were captured using parameters such as walking speed, cadence, and the 6-Minute Walk Test. This comprehensive mapping of outcomes and assessment tools supports the conclusion that balance, particularly static balance, remains the most consistently evaluated domain across systematic reviews likely due to its clinical relevance in fall prevention among individuals with diabetic peripheral neuropathy.

Physical exercise is crucial for overall health, and in patients with diabetes, it improves glycemic control, reduces cardiovascular risk, aids in weight management, and enhances well-being [66, 73, 74], Specifically, in patients with DPN, physical training can be an effective intervention for many associated symptoms [75], including pain. Although physical exercise interventions are considered crucial in the treatment of diabetic patients with DPN, with or without ulcers, protective strategies are often preferred over therapeutic exercises, which can have unforeseen consequences in these patients [76], especially those at high risk of ulceration. To avoid complications from exercise training, it is necessary to provide evidence-based recommendations on the benefits and potential harms of exercise therapy and to understand the most important safety measures when recommending exercises to patients with DPN to minimize risks.

Recently, a guideline was published with a series of recommendations obtained through an international and multidisciplinary Delphi consensus on physical exercise in diabetic patients at risk of foot ulceration. The aim was to structure the types and recommendations for patients with different levels of ulceration risk to have guidelines when prescribing and performing physical exercise [77]. It is important to consider these recommendations when planning a therapeutic exercise program for this type of patient.

The modalities of exercise programs varied, with many conducted in groups and some at home with telephone follow-up. The literature highlights the advantages of group exercise for motivation and adherence, recommending continuous and personalized support, and the inclusion of group activities to enhance motivation [77–79] Regarding the duration and frequency of the studied interventions, they are consistent with the recommendations of the ADA [58].

In general, the results and conclusions of the individual studies included in the systematic reviews on the impact of exercise training in patients with DPN should be interpreted with caution, as their methodological limitations, such as small sample sizes (N < 100), can affect both the internal and external validity of their results. Therefore, the conclusions of the reviews found to date are not definitive and require confirmation through studies with greater methodological rigor and larger sample sizes. Future reviews should aim to group results based on the methodological quality of the selected articles to answer research questions with the highest possible level of evidence.

Thus, despite the large number of interventions included in this review, a high level of evidence in the obtained results cannot be guaranteed. Additionally, there was significant variability in health outcomes and different types of tools used to measure each of them, which complicates providing solid evidence on the effectiveness of these interventions. The meta-analyses provided by some reviews show high heterogeneity among the included studies, meaning the overall reported effect may not adequately represent individual effects. Future research should focus on the health outcomes most relevant for implementing a physical exercise intervention in patients with DPN, including safety evaluation and using measurement tools previously agreed upon by experts.

Another limitation of these systematic reviews included is the lack of differentiation between patients with T1DM and T2DM, as well as between obese and non-obese individuals. The included systematic reviews focused on patients diagnosed with DPN as a whole, without stratifying by diabetes type or BMI status. As a result, the findings of this review cannot determine whether the effects of exercise therapy differ between these subgroups. Future studies should aim to investigate whether specific exercise interventions may have varying impacts on patients with T1DM versus T2DM or on obese versus non-obese individuals. Understanding these potential differences could contribute to more tailored and effective exercise programs for individuals with DPN.

According to AMSTAR-2 criteria, the systematic reviews found on the benefits of exercise training on different outcomes in patients with DPN are not reliable or may not provide an accurate and comprehensive summary of the available studies. Specifically, regarding whether the review authors explain why they include a particular type of study (item 3), none of the reviews fulfill this requirement because it is assumed to be understood with the type of question posed. Therefore, this aspect is not considered to reduce the quality of the review.

The search strategy is not usually completely exhaustive in any of the reviews, which could be due to the lack of interest in searching sources other than scientific databases. It is recommended that future reviews include the reason for not conducting a search in all types of sources. Another frequently unmet item is item 7, as most reviews list the reasons for exclusion but do not provide a list of excluded documents because this procedure is not yet sufficiently established. Often, in reviews that include meta-analyses, the causes of heterogeneity are not analyzed when there is high heterogeneity, making it difficult to trust the results of these meta-analyses. Publication bias is also not analyzed in most systematic reviews. In none of the meta-analyses reviewed in this umbrella Review, except for one, was publication bias assessed, which confirms the high risk of bias in these reviews, requiring us to be very cautious when interpreting their results. Lastly, it is uncommon for a systematic review to report the sources of funding for the included studies (item 10).

When evaluating the level of confidence, all reviews resulted in low and very low confidence. However, if we do not consider item 7 as a critical domain (by viewing it more as a reporting error than a risk of bias since the reasons for exclusion are at least reported), two of the reviews [27, 28] could be considered to have low rather than very low confidence.

Regarding the limitations of this umbrella review, despite consulting five databases, there is a risk that reviews present in other unexamined databases were not included. Although grey literature was not considered, and some relevant insights could be missing, it is unlikely that systematic reviews in this field are published there.

Additionally, this review did not collect information on the funding sources of the included reviews, as it was considered not to be a relevant element given that the reviews focused on evaluating exercise training interventions, thereby excluding the influence of the pharmaceutical industry or other potential interests.

Although numerous systematic reviews have explored the effects of exercise in patients with DPN, the current evidence remains fragmented, heterogeneous, and often of low methodological quality. This umbrella review contributes by synthesizing data from individual studies, applying consistent eligibility criteria, and categorizing interventions by exercise modality to map their impact across diverse health outcomes. Thus, we provide clearer and more clinically useful insights into which types of exercise are most consistently associated with improvements in balance, glycemic control, neuropathic symptoms, or physical function. This synthesis supports evidence-based decision-making and highlights key research gaps. The fact that many of the included reviews were of low or very low methodological quality further reinforces the need for well-designed future studies and reviews. Our approach, based on a structured qualitative synthesis of primary studies, offers a more transparent and rigorous summary of the available evidence, minimizing the limitations of previous reviews.

## Conclusions

This umbrella review provides a comprehensive overview of the effects of exercise training on health outcomes in patients with DPN. Despite considerable variability in exercise modalities and outcome measures, and the overall low methodological quality of the included reviews, the evidence suggests that exercise, particularly when combining aerobic, strength, and balance training, can improve glycemic control, reduce neuropathic symptoms, enhance joint mobility, and support physical function. However, the effects on other outcomes, such as fall risk and ulcer prevention, remain inconclusive. In addition, this umbrella review identified a wide range of assessment tools used across studies, highlighting the lack of standardization in outcome measurement. This inconsistency limits comparability and may contribute to conflicting results in the literature. Future research should aim for greater methodological rigor and uniformity in outcome selection and measurement. It is also important to explore the differential impact of exercise interventions according to diabetes type and to develop safe, tailored exercise protocols that minimize the risk of complications, such as foot ulceration.

## Supplementary Information

Additional file 1.

Additional file 2.

Additional file 3.

Additional file 4.

## Data Availability

All necessary information to support the data reported in this study is available in the supplementary materials of this publication. No new datasets were generated during the study, and the data used are fully described and accessible in the attached supplementary files.
