# Supplementary material for: Impact of Exercise Training in Patients with Diabetic Peripheral Neuropathy: An Umbrella Review
Source: Sports Med Open. 2025 Jun 15;11:75. doi: 10.1186/s40798-025-00863-4 (PMC12167735; doi:10.1186/s40798-025-00863-4)
Supplement: Supplementary file 1 — Additional file 1. [file 40798_2025_863_MOESM1_ESM.docx]

**Supplementary Material 1.** Search strategy for each database.

**Date of search:** September 11, 2023

**Database: COCHRANE LIBRARY**

n=78

**#1** (Diabetes):ti,ab,kw OR (Diabetic mellitus):ti,ab,kw OR (Diabetic patients):ti,ab,kw OR (Diabetic Neuralgia*):ti,ab,kw

(Word variations have been searched)S Limits

n=115708

**#2** (Complication*):ti,ab,kw OR (Arteriopath*):ti,ab,kw OR (Foot):ti,ab,kw OR (Feet):ti,ab,kw OR (Neuropath*):ti,ab,kw

(Word variations have been searched)S Limits

n=257199

**#3** (ulcer*):ti,ab,kw

(Word variations have been searched)S Limits

n=31370

**#4** (Exercise*):ti,ab,kw OR (Physical Activity):ti,ab,kw OR (Proprioceptive):ti,ab,kw OR (rehabilitation intervention):ti,ab,kw OR (rehabilitation interventions):ti,ab,kw

(Word variations have been searched)S Limits

n=194082

**#5** (Stretching):ti,ab,kw OR (Walking):ti,ab,kw OR (Training):ti,ab,kw OR (Physical Activities):ti,ab,kw OR (Physical Fitness):ti,ab,kw

(Word variations have been searched)S Limits

n=225188

**#6** (Cycling):ti,ab,kw OR (Sport):ti,ab,kw OR (Running):ti,ab,kw

(Word variations have been searched)S Limits

n=105946

**#7** #2OR#3

Limits

n=281086

**#8** #4OR#5OR#6

Limits

n=367026

**#9** #1AND#7AND#8

Limits

n=5238

**78 Cochrane Reviews matching "#9 - #1AND#7AND#8"**

**Database: EMBASE**

n=303

**#1** 'diabetes mellitus'/exp OR 'diabetes mellitus' OR diabetes:ti,ab OR 'diabetic mellitus':ti,ab OR 'diabetic patients':ti,ab OR 'diabetic neuralgia*':ti,ab

n=1,445,942

**#2** 'exercise'/exp OR 'exercise' OR 'stretching exercise'/exp OR 'stretching exercise' OR 'sport'/exp OR 'sport' OR exercise*:ti,ab OR 'physical activity':ti,ab OR proprioceptive:ti,ab OR 'rehabilitation interventions':ti,ab OR 'rehabilitation intervention':ti,ab OR stretching:ti,ab OR walking:ti,ab OR training:ti,ab OR 'physical activities':ti,ab OR 'physical fitness':ti,ab OR cycling:ti,ab OR sport:ti,ab OR running:ti,ab

n=[1,881,271](https://www.embase.com/)

**#3** 'diabetic complication'/exp OR 'diabetic complication' OR 'diabetic neuropathy'/exp OR 'diabetic neuropathy' OR 'diabetic foot'/exp OR 'diabetic foot' OR 'foot ulcer'/exp OR 'foot ulcer' OR 'foot disease'/exp OR 'foot disease' OR complication*:ti,ab OR arteriopath*:ti,ab OR foot:ti,ab OR feet:ti,ab OR ulcer*:ti,ab OR neuropath*:ti,ab

n=[2,506,656](https://www.embase.com/)

**#4** #1 AND #2 AND #3

n=[18,286](https://www.embase.com/)

**#5** #4 AND 'review'/it AND ('meta analysis topic'/de OR 'systematic review'/de OR 'systematic review topic'/de)

n=[390](https://www.embase.com/)

**#6** #5 AND [embase]/lim NOT ([embase]/lim AND [medline]/lim)

n=102

**Database: PubMed**

**#1** Search: ((((Diabetes Mellitus[MeSH Terms]) OR (Diabetes[Title/Abstract])) OR ("Diabetic mellitus"[Title/Abstract])) OR ("Diabetic patients"[Title/Abstract])) OR ("Diabetic Neuralgia"[Title/Abstract])

**#2** Search: ((((((((((((((Exercise[MeSH Terms]) OR (Muscle Stretching Exercises[MeSH Terms])) OR (Sport[MeSH Terms])) OR (Exercise*[Title/Abstract])) OR ("Physical Activity"[Title/Abstract])) OR (Proprioceptive[Title/Abstract])) OR ("rehabilitation interventions"[Title/Abstract]) OR ("rehabilitation intervention"[Title/Abstract])) OR (Stretching[Title/Abstract])) OR (Walking[Title/Abstract])) OR (Training[Title/Abstract]) ) OR ("Physical Activities"[Title/Abstract])) OR (Physical Fitness[Title/Abstract])) OR (Cycling[Title/Abstract])) OR (Sport[Title/Abstract])) OR (Running[Title/Abstract])

**#3** Search: (((((((((((Diabetes Complications[MeSH Terms]) OR (Diabetic Neuropathies[MeSH Terms])) OR (diabetic foot[MeSH Terms])) OR (Foot[MeSH Terms])) OR (foot ulcer[MeSH Terms])) OR (Foot Diseases[MeSH Terms])) OR (Complication*[Title/Abstract])) OR (Arteriopath*[Title/Abstract])) OR (Foot[Title/Abstract])) OR (Feet[Title/Abstract])) OR (Ulcer*[Title/Abstract])) OR (Neuropath*[Title/Abstract])

Search:

#1 AND #2 AND #3 Filters: Meta-Analysis, Systematic Review

n=303

**Database: SCOPUS**

n=695

( ( TITLE-ABS-KEY ( diabetes ) OR TITLE-ABS-KEY ( diabetic AND mellitus ) OR TITLE-ABS-KEY ( diabetic AND patients ) OR TITLE-ABS-KEY ( diabetic AND neuralgia* ) ) ) AND ( ( TITLE-ABS-KEY ( exercise* ) OR TITLE-ABS-KEY ( physical AND activity ) OR TITLE-ABS-KEY ( proprioceptive ) OR TITLE-ABS-KEY ( rehabilitation AND interventions ) OR TITLE-ABS-KEY ( rehabilitation AND intervention ) OR TITLE-ABS-KEY ( stretching ) OR TITLE-ABS-KEY ( walking ) OR TITLE-ABS-KEY ( training ) OR TITLE-ABS-KEY ( physical AND activities ) OR TITLE-ABS-KEY ( physical AND fitness ) OR TITLE-ABS-KEY ( cycling ) OR TITLE-ABS-KEY ( sport ) OR TITLE-ABS-KEY ( running ) ) ) AND ( ( TITLE-ABS-KEY ( complication* ) OR TITLE-ABS-KEY ( diabetes AND complications ) OR TITLE-ABS-KEY ( diabetic AND neuropathies ) OR TITLE-ABS-KEY ( diabetic AND foot ) OR TITLE-ABS-KEY ( foot ) OR TITLE-ABS-KEY ( foot AND ulcer ) OR TITLE-ABS-KEY ( foot AND diseases ) OR TITLE-ABS-KEY ( complication* ) OR TITLE-ABS-KEY ( arteriopath* ) OR TITLE-ABS-KEY ( foot ) OR TITLE-ABS-KEY ( feet ) OR TITLE-ABS-KEY ( neuropath* ) ) ) AND ( TITLE-ABS-KEY ( "systematic review" ) ) AND ( LIMIT-TO ( SRCTYPE , "j" ) ) AND ( LIMIT-TO ( DOCTYPE , "re" ) )

695 documents found

**Database: SPORT DISCUSS**

n=27

| **#** | **Consult** | **Limiters and Expanders** | **Last accessed through** | **Results** |
| --- | --- | --- | --- | --- |
| S16 | (S9 AND S10 AND S11) AND TI review | Expanders - Apply Equivalent Subjects  Search Modes - Boolean/Phrase | Interfaz - EBSCOhost Research Databases Pantalla de búsqueda - Búsqueda avanzada Base de datos - SPORTDiscus with Full Text | 27 |
| S15 | S9 AND S10 AND S11 | Limiters - Publication Date: -20230431; Publication Type:Review Expanders - Apply Equivalent Subjects  Search Modes - Boolean/Phrase | Interface - EBSCOhost Research Databases  Search Screen - Advanced Search  Database - SPORTDiscus with Full Text | 0 |
| S14 | S9 AND S10 AND S11 | Limiters - Publication Date: -20230431; Publication Type: Review; Document Type: Article Expanders - Apply Equivalent Subjects  Search Modes - Boolean/Phrase Búsqueda en SmartText | Interface - EBSCOhost Research Databases  Search Screen - Advanced Search  Database - SPORTDiscus with Full Text | 0 |
| S13 | S9 AND S10 AND S11 | Limiters - Publication Date: -20230431; Publication Type: Review; Document Type: Article Expanders - Apply Equivalent Subjects  Search Modes - Boolean/Phrase | Interface - EBSCOhost Research Databases  Search Screen - Advanced Search  Database - SPORTDiscus with Full Text | 0 |
| S12 | S9 AND S10 AND S11 | Expanders - Apply Equivalent Subjects  Search Modes - Boolean/Phrase | Interface - EBSCOhost Research Databases  Search Screen - Advanced Search  Database - SPORTDiscus with Full Text | 516 |
| S11 | S4 OR S5 | Expanders - Apply Equivalent Subjects  Search Modes - Boolean/Phrase | Interface - EBSCOhost Research Databases  Search Screen - Advanced Search  Database - SPORTDiscus with Full Text | 51,232 |
| S10 | S2 OR S3 OR S6 OR S7 | Expanders - Apply Equivalent Subjects  Search Modes - Boolean/Phrase | Interface - EBSCOhost Research Databases  Search Screen - Advanced Search  Database - SPORTDiscus with Full Text | 660,639 |
| S9 | S1 OR S8 | Expanders - Apply Equivalent Subjects  Search Modes - Boolean/Phrase | Interface - EBSCOhost Research Databases  Search Screen - Advanced Search  Database - SPORTDiscus with Full Text | 17,069 |
| S8 | TI Diabetes OR TI diabetic mellitus OR TI diabetic patients OR TI Diabetic Neuralgia* | Expanders - Apply Equivalent Subjects  Search Modes - Boolean/Phrase | Interface - EBSCOhost Research Databases  Search Screen - Advanced Search  Database - SPORTDiscus with Full Text | 6,782 |
| S7 | TI Running | Expanders - Apply Equivalent Subjects  Search Modes - Boolean/Phrase | Interface - EBSCOhost Research Databases  Search Screen - Advanced Search  Database - SPORTDiscus with Full Text | 17,630 |
| S6 | TI Exercise* OR TI Physical Activity OR TI Proprioceptive OR TI rehabilitation interventions OR TI rehabilitation intervention OR TI Stretching OR TI Walking OR TI Training OR TI Physical Activities OR TI Physical Fitness OR TI Cycling OR TI Sport | Expanders - Apply Equivalent Subjects  Search Modes - Boolean/Phrase | Interface - EBSCOhost Research Databases  Search Screen - Advanced Search  Database - SPORTDiscus with Full Text | 310,232 |
| S5 | TI Complication* OR TI Foot OR TI Arteriopath* OR TI Feet OR TI Ulcer* OR TI Neuropath* | Expanders - Apply Equivalent Subjects  Search Modes - Boolean/Phrase | Interface - EBSCOhost Research Databases  Search Screen - Advanced Search  Database - SPORTDiscus with Full Text | 12,674 |
| S4 | AB Complication* OR AB Foot OR AB Arteriopath* OR AB Feet OR AB Ulcer* OR AB Neuropath* | Expanders - Apply Equivalent Subjects  Search Modes - Boolean/Phrase | Interface - EBSCOhost Research Databases  Search Screen - Advanced Search  Database - SPORTDiscus with Full Text | 46,090 |
| S3 | AB Running | Expanders - Apply Equivalent Subjects  Search Modes - Boolean/Phrase | Interface - EBSCOhost Research Databases  Search Screen - Advanced Search  Database - SPORTDiscus with Full Text | 46,953 |
| S2 | AB Exercise* OR AB Physical Activity OR AB Proprioceptive OR AB rehabilitation interventions OR AB rehabilitation intervention OR AB Stretching OR AB Walking OR AB Training OR AB Physical Activities OR AB Physical Fitness OR AB Cycling OR AB Sport | Expanders - Apply Equivalent Subjects  Search Modes - Boolean/Phrase | Interface - EBSCOhost Research Databases  Search Screen - Advanced Search  Database - SPORTDiscus with Full Text | 484,047 |
| S1 | AB diabetes OR AB diabetes mellitus OR AB diabetic patients OR AB Diabetic Neuralgia* | Expanders - Apply Equivalent Subjects  Search Modes - Boolean/Phrase | Interface - EBSCOhost Research Databases  Search Screen - Advanced Search  Database - SPORTDiscus with Full Text | 15,579 |
