# Supplementary material for: Impact of Exercise Training in Patients with Diabetic Peripheral Neuropathy: An Umbrella Review
Source: Sports Med Open. 2025 Jun 15;11:75. doi: 10.1186/s40798-025-00863-4 (PMC12167735; doi:10.1186/s40798-025-00863-4)
Supplement: Supplementary file 2 — Additional file 2. [file 40798_2025_863_MOESM2_ESM.pdf]

Impact of exercise training in patients with diabetic peripheral neuropathy: An Umbrella Review

Alba Gracia-Sánchez [1], Adriana López-Pineda [2], Rauf Nouni-García [3], Sara Zúnica-García [4], Esther Chicharro-Luna[5*] and Vicente F Gil-Guillén [6*]

[1] Behavioral Sciences and Health Department. Nursing Area. Faculty of Medicine. Miguel Hernandez University, San Juan de Alicante, Spain. ORCID: 0000-0001-9643-4548.

[2] Clinical Medicine Department, Miguel Hernandez University, San Juan de Alicante, Spain; Network for Research on Chronicity, Primary Care, and Health Promotion (RICAPPS), Alicante, Spain. ORCID: 0000-0002-2117-0178. Email: adriana.lopezp@umh.es

[3] Pathology and Surgery Department, Miguel Hernandez University, San Juan de Alicante, Spain; Network for Research on Chronicity, Primary Care, and Health Promotion (RICAPPS), Alicante, Spain; Institute of Health and Biomedical Research of Alicante (ISABIAL), Alicante, Spain.

[4] Behavioral Sciences and Health Departament. Nursing Area. Faculty of Medicine. Miguel Hernandez University, San Juan de Alicante, Spain. ORCID: 0000-0001-6657-7636.

[5] Behavioral Sciences and Health Department. Nursing Area. Faculty of Medicine. Miguel Hernandez University, San Juan de Alicante, Spain. Institute of Health and Biomedical Research of Alicante (ISABIAL). Spain. ORCID: 0000-0003-4766-5723.

[6] Clinical Medicine Department, Miguel Hernandez University, San Juan de Alicante, Spain; Network for Research on Chronicity, Primary Care, and Health Promotion (RICAPPS), Alicante, Spain; Research Unit, University General Hospital of Elda, Elda, Spain.

*These authors contributed equally to this work and share senior authorship.

Supplementary Material 2. Excluded articles after full text review.

References of Excluded articles	Reason for exclusion
Pedersen BS, Kodal LS, Kaalund AB, Holm-Yildiz S, Pedersen MM, Dysgaard T. Effect of strength training on functional outcomes and strength in patients with polyneuropathy: A scoping review. Front Physiol. 2023 Apr 6;14:1158039. doi: 10.3389/fphys.2023.1158039. PMID: 37089431; PMCID: PMC10116572.	Wrong study design
Albalawi H, Coulter E, Ghouri N, Paul L. The effectiveness of structured exercise in the south Asian population with type 2 diabetes: a systematic review. Phys Sportsmed. 2017 Nov;45(4):408-417. doi: 10.1080/00913847.2017.1387022. Epub 2017 Oct 13. PMID: 28971713.	Wrong patient population (only 2 articles speak about neuropathy)
Rietz M, Lehr A, Mino E, Lang A, Szczerba E, Schiemann T, Herder C, Saatmann N, Geidl W, Barbaresko J, Neuenschwander M, Schlesinger S. Physical Activity and Risk of Major Diabetes-Related Complications in Individuals With Diabetes: A Systematic Review and Meta-Analysis of Observational Studies. Diabetes Care. 2022 Dec 1;45(12):3101-3111. doi: 10.2337/dc22-0886. PMID: 36455117; PMCID: PMC9862380.	Wrong patient population
Streckmann F, Zopf EM, Lehmann HC, May K, Rizza J, Zimmer P, Gollhofer A, Bloch W, Baumann FT. Exercise intervention studies in patients with peripheral neuropathy: a systematic review. Sports Med. 2014 Sep;44(9):1289-304. doi: 10.1007/s40279-014-0207-5. PMID: 24927670.	Wrong patient population
Davies B, Cramp F, Gauntlett-Gilbert J, Wynick D, McCabe CS. The role of physical activity and psychological coping strategies in the management of painful diabetic neuropathy--A systematic review of the literature. Physiotherapy. 2015 Dec;101(4):319-26. doi: 10.1016/j.physio.2015.04.003. Epub 2015 Apr 22. PMID: 26036692.	Wrong intervention
Liao F, An R, Pu F, Burns S, Shen S, Jan YK. Effect of Exercise on Risk Factors of Diabetic Foot Ulcers: A Systematic Review and Meta-Analysis. Am J Phys Med Rehabil. 2019 Feb;98(2):103-116. doi: 10.1097/PHM.0000000000001002. PMID: 30020090.	Wrong patient population
Medeiros S, Rodrigues A, Costa R. Physiotherapeutic interventions in the treatment of patients with diabetic foot ulcers: a systematic literature review. Physiotherapy. 2023 Mar;118:79-87. doi: 10.1016/j.physio.2022.09.006. Epub 2022 Sep 17. PMID: 36244842.	Wrong patient population
Tran MM, Haley MN. Does exercise improve healing of diabetic foot ulcers? A systematic review. J Foot Ankle Res. 2021 Mar 20;14(1):19. doi: 10.1186/s13047-021-00456-w. PMID: 33743791; PMCID: PMC7980337.	Wrong patient population (pacient have a ulcers lesions)

van Netten JJ, Fijen VM, Bus SA. Weight-bearing physical activity in people with diabetes-related foot disease: A systematic review. Diabetes Metab Res Rev. 2022 Sep;38(6):e3552. doi: 10.1002/dmrr.3552. Epub 2022 Jun 13. PMID: 35668034; PMCID: PMC9539904.	Wrong intervention
Carvajal-Moreno L, Coheña-Jiménez M, García-Ventura I, Pabón-Carrasco M, Pérez-Belloso AJ. Prevention of Peripheral Distal Polyneuropathy in Patients with Diabetes: A Systematic Review. J Clin Med. 2022 Mar 21;11(6):1723. doi: 10.3390/jcm11061723. PMID: 35330052; PMCID: PMC8948704.	Wrong intervention
Ites KI, Anderson EJ, Cahill ML, Kearney JA, Post EC, Gilchrist LS. Balance interventions for diabetic peripheral neuropathy: a systematic review. J Geriatr Phys Ther. 2011 Jul-Sep;34(3):109-16. doi: 10.1519/JPT.0b013e318212659a. PMID: 21937901.	Wrong intervention
Fuller, A.A., Singleton, J.R., Smith, A.G. and Marcus, R.L. (2016) Exercise in Type 2 Diabetic Peripheral Neuropathy. Current Geriatrics Reports, 5, 150-159. https://doi.org/10.1007/s13670-016-0177-6	Wrong study design
Wendland DM, Kline PW, Simmons L, Sinacore DR. The effect of exercise, physical activity, stepping characteristics, and loading on diabetic foot ulcer healing: a systematic review. Wounds. 2023 Jan;35(1):9-17. doi: 10.25270/wnds/22007. PMID: 36749703.	Wrong intervention
Liampas A, Hadjigeorgiou L, Nteveros A, Ioannou C, Varrassi G, Zis P. Adjuvant physical exercise for the management of painful polyneuropathy. Postgrad Med. 2022 Jun;134(5):458-462. doi: 10.1080/00325481.2021.2004733. Epub 2021 Nov 26. PMID: 34779342.	Wrong patient population
Zilliox LA, Russell JW. Physical activity and dietary interventions in diabetic neuropathy: a systematic review. Clin Auton Res. 2019 Aug;29(4):443-455. doi: 10.1007/s10286-019-00607-x. Epub 2019 May 10. PMID: 31076938; PMCID: PMC6697618.	Wrong intervention
Faizah, R., Efendi, F., & Suprajitno, S. (2020). A Systematic Review of Foot Exercises with Group Support to Improve the Foot Health of Diabetes Mellitus Patients. Jurnal Ners, Special Issues, 129-134. doi:http://dx.doi.org/10.20473/jn.v15i2.18996	Wrong patient population
Hou L, Wang Q, Pan B, Li R, Li Y, He J, Qin T, Cao L, Zhang N, Cao C, Ge L, Yang K. Exercise modalities for type 2 diabetes: A systematic review and network meta-analysis of randomized trials. Diabetes Metab Res Rev. 2023 Jan;39(1):e3591. doi: 10.1002/dmrr.3591. Epub 2022 Dec 4. PMID: 36423199.	Wrong patient population
Orlando G, Balducci S, Boulton AJM, Degens H, Reeves ND. Neuromuscular dysfunction and exercise training in people with diabetic peripheral neuropathy: A narrative review. Diabetes Res Clin Pract. 2022 Jan;183:109183. doi: 10.1016/j.diabres.2021.109183. Epub 2021 Dec 17. PMID: 34929255.	Wrong study design
Aagaard TV, Moeini S, Skou ST, Madsen UR, Brorson S. Benefits and Harms of Exercise Therapy for Patients With Diabetic Foot Ulcers: A Systematic Review. Int J Low Extrem Wounds. 2022 Sep;21(3):219-233. doi: 10.1177/1534734620954066. Epub 2020 Sep 14. PMID: 32924691.	Wrong patient population

Streckmann F, Balke M, Cavaletti G, Toscanelli A, Bloch W, Décard BF, Lehmann HC, Faude O. Exercise and Neuropathy: Systematic Review with Meta-Analysis. Sports Med. 2022 May;52(5):1043-1065. doi: 10.1007/s40279-021-01596-6. Epub 2021 Dec 29. PMID: 34964950.	Wrong patient population
Riddell MC, Burr J. Evidence-based risk assessment and recommendations for physical activity clearance: diabetes mellitus and related comorbidities. Appl Physiol Nutr Metab. 2011 Jul;36 Suppl 1:S154-89. doi: 10.1139/h11-063. PMID: 21800941.	Diabetes, without NP (Wrong patient)
Jones K, Backhouse MR, Bruce J. Rehabilitation for people wearing offloading devices for diabetes-related foot ulcers: a systematic review and meta-analyses. J Foot Ankle Res. 2023 Mar 25;16(1):16. doi: 10.1186/s13047-023-00614-2. PMID: 36966316; PMCID: PMC10039553.	Wrong intervention
van Laake-Geelen CCM, Smeets RJEM, Quadflieg SPAB, Kleijnen J, Verbunt JA. The effect of exercise therapy combined with psychological therapy on physical activity and quality of life in patients with painful diabetic neuropathy: a systematic review. Scand J Pain. 2019 Jul 26;19(3):433-439. doi: 10.1515/sjpain-2019-0001. PMID: 31112511.	Wrong intervention
